# Supplementary material for: State of Knowledge on the Acquisition, Diversity, Interspecies Attribution and Spread of Antimicrobial Resistance between Humans, Animals and the Environment: A Systematic Review
Source: Antibiotics (Basel). 2022 Dec 31;12(1):73. doi: 10.3390/antibiotics12010073 (PMC9854550; doi:10.3390/antibiotics12010073)
Supplement: Supplementary file 1 [file antibiotics-12-00073-s001.zip › antibiotics-1939220-Online Supplement S1.pdf]

Online Supplement S1

TABLE OF CONTENT

ANALYSIS PLAN SYSTEMATIC REVIEW 4
1. Dataset: *Number of publications, methods and criteria of selection, classification* *4*
A) Search criteria for basic dataset 4
Search terms and database 4
Time range for search 4
B) Methods and criteria of selection 4
Step 1: Publications investigating two out of three compartments 4
Step 2: Publications investigating all three compartments 5
Step 3: Finding consensus 6
Step 4: Correction of consensus 6
C) Numbers of sorted publications 7
Step 1: Publications investigating two out of three compartments 7
Step 2: Publications investigating all three compartments 7
Step 3: Finding consensus 8
Step 4: Correction of consensus 8
Exclusions after analysis of observed compartments (environment, animal, human) (Step 4) 9
D) Papers included in Systematic Review 12
2. Hermeneutic literature analysis *16*
Established Code Groups and Codes 16
3. Investigated fields: *Which animals, environments and humans are known so far? (Animal species, type of environment, geographical location (country))* *28*
Ecosystem interactions 28
Animal species 28
Type of environment 29
Geographical representation 30
Human population 31
4. Investigated bacteria and resistance genes: *Which bacteria (taxonomy) and resistance genes were investigated? Which classes of antimicrobial agents were investigated in the resistance testing?* *32*
Gram-negative bacteria 32
Gram-positive bacteria 32
Gram-indeterminate bacteria 32
Summary of investigated phyla of bacteria 33
Overview of bacteria represented in included studies 33
Classification of antibiotic resistances 36
Phenotypic resistance: Antimicrobials investigated in included studies 36
Genotypic resistance: Classification of mobile genetic elements and antimicrobial resistance genes and their encoding resistance represented in included studies 41
Genotypic resistance: Antimicrobial resistance genes represented in included studies (quantitative analysis) ... 49
Number of studies investigating phenotypic or/and genotypic resistance 57
5. Methods for antimicrobial susceptibility testing: *Phenotypic vs. genotypic resistance, PCR, metagenomic study, whole genome sequencing, MIC, …* *58*
Phenotypic resistance 58
Genotypic resistance 59
6. Methods for investigation of the direction of transmission/spread: *phylogeny, …* *59*
APPENDIX 60
Appendix I: List of analysis methods for antimicrobial resistance in studies included *60*
Phenotypic resistance 60
Genotypic resistance 60
Appendix II: List of additional analysis methods for antimicrobial resistance in studies included *62*

LIST OF TABLES

Table S1. Distribution of papers in EndNote library in step 1. 7
Table S2. Distribution of papers in EneNote library in step 2. 8
Table S3. Distribution of papers in EndNote library in step 3. 8
Table S4. Distribution of papers in EndNote library step 4. 9
Table S5. Reasons for correction of consensus and exclusion of papers in step 4.................. 11
Table S6. Established Code Groups and Codes in Atlas.ti8.. 27
Table S7. Overview of bacteria represented in studies included.. 35
Table S8. Antimicrobials investigated in included studies (n).. 40
Table S9. Antibiotic resistance genes (ARGs) investigated in included studies and corresponding resistance. 48
Table S10. Mobile genetic elements investigated in included studies. 49
Table S11. Antibiotic resistance genes (ARGs) investigated in included studies (n) and corresponding resistance. 56
Table S12. Mobile genetic elements investigated in included studies (n)............................... 57
Table S13. Methods for Phenotypic antimicrobial susceptibility testing................................ 58

LIST OF FIGURES

Figure S1. Types of animal sectors investigated in studies included in our systematic review (number of studies (n)).. 28
Figure S2. Types of animal products/food of animal origin represented in the 41 studies included in our systematic review investigating this compartment (number of studies (n)). .. 29
Figure S3. Environmental niches represented in our systematic review (number of studies (n)).. 29
Figure S4. Types of water sources represented in the 60 studies investigating water environment included in our systematic review... 30
Figure S5. Countries represented in studies included in our systematic review. 30
Figure S6. Countries only represented once in included studies are summarized in group “other”. 31
Figure S7. Human compartment divided into clinical, non-clinical and unknown................. 31
Figure S8. Bacteria genera investigated in studies (n). 33
Figure S9. Bacteria phyla represented in studies (n)... 33

Analysis Plan Systematic Review

In this Systematic Review we intended to investigate, systematically analyze and summarize the knowledge on the circulation of antibiotic resistant bacteria and resistance genes (named "resistome" as an entity).

1. Dataset: Number of publications, methods and criteria of selection, classification

A) Search criteria for basic dataset

Search terms and database

The analysis is based on the following search strategy applied in the **PubMed** database on the 12.3.2019:

((resistome OR resistomes OR antibiotic resistance gene OR antibiotic resistance genes OR antibiotic resistan* OR microbial resistan*)) AND ((animal AND human) OR (animal AND environment) OR (human AND environment))

searching in "all fields".

The authors decided to limit their search on the PubMed database due to the amount of hits achieved using these search criteria. *For a comprehensive systematic review we may should have searched other databases.*

Time range for search

The search was limited to publication dates from 01.01.2004 to 12.03.2019. We have chosen this time period because since the year 2004 the number of publications on this topic began to increase significantly and it was a realistic time range to investigate (workload, knowledge, etc.).

B) Methods and criteria of selection

Proceeding for selection of papers to be included in our work was according to the PRISMA guidelines on the writing of systematic reviews. The application used for search and selection of papers was EndNote X9.2. Two students (Keira Spinner, ETH Zurich and Hélène Meier, University of Berne) completed the search and selection of papers using the full database (9115 publications) and found consensus on the 20.04.2020. Prof. Jakob Zinsstag and Lisa Crump examined this consensus dataset on a random basis.

Classification of the basic dataset was divided into two steps:

1. Step 1: Rough sorting of publications useful for our work fulfilling the criteria fitting OneHealth approach on antibiotic resistance in bacteria
2. Step 2: Further sorting of publications in the folder "Inclusion_def" fulfilling the criteria of investigating the fields human, environment <u>and</u> animal concerning OneHealth approach on antibiotic resistance in bacteria
3. Step 3: Finding consensus between Keira Spinner and Hélène Meier out of their file "3 criteria inclusion"
4. Step 4: Correcting consensus while Hélène Meier read the papers for writing this analysis plan and finding consensus with Keira Spinner on those papers. Opening new file "3 criteria inclusion consensus korr"

Step 1: Publications investigating two out of three compartments

Sorting of Publications fitting OneHealth approach on antibiotic resistance, meaning: At least two of the three compartments (animal-environment-human) were investigated/sampled in the publication and the publication is investigating antimicrobial resistance in

bacteria/antimicrobial resistance genes. The publication provides information about resistance/resistance genes carriage shared by either animals and humans or animals and environment or environment and humans. Additionally, the bacteria which are transmitted in the ecosystem are studied in the included articles. A further (but not mandatory) criteria is the analysis of the resistance genes transmission/spread.
Divison into following categories:

- "Access not working": No access with VPN media player
- "Duplicates": There should have been no duplicates in EndNote since PubMed recognizes them.
- "Exclusion_def": Publications not fitting our OneHealth approach that are therefore excluded for our review
- "Inclusion_def": Publications fitting our OneHealth approach/selection criteria
- "Inclusion_evtl": Publications investigating soil contamination, food contamination (e.g. milk/cheese), water contamination, slaughterhouses and its environment, wild life animal contamination, sewage analysis (e.g. hospital wastewater), livestock animal buildings (e.g. pig/poultry) not exactly fitting our OneHealth approach or only in a broader sense.
- "Ø Language": Publications in languages that the authors do not master*
- "Ø Language but important": Publications in languages that the authors do not master*, but that seem to fit our criteria (OneHealth approach)
- "OtherFormatNotRelevant": Publication of another format than a study (e.g. letter to editor, perspective, review,…) that is not relevant for our systematic review
- "Retracted article": One article was retracted during the period of writing this systematic review
- "TextsForSources/otherformat": Publication of another format than a study (e.g. letter to editor, perspective, review,…) that is relevant for our systematic review and could be used as a reference or to check if the most relevant papers were included

Keira Spinner still included publications of other format (e.g. reviews) in the first selection process in order to conduct the convenient snow-balling technique, Hélène Meier already sorted them out into another folder ("TextsForSources/otherformat" or "OtherFormatNotRelevant").
Publications in languages that the authors do not master were collected in separate folders ("øLanguage" or "øLanguage but important"). Authors master and therefore included publications of the following languages: German, English, French, (Spanish, Italian).
It was a common decision to only include papers generating (primary) data and investigating isolates for their resistance patterns. We therefore decided to exclude theoretical scenarios (e.g. epidemiological modeling) or other formats (e.g. study protocols, reviews, reports).
Papers investigating all three compartments where the environmental sector is closely related to the animal and human sector were excluded, e.g. papers investigating direct environment (litter samples, feeding installations, etc.) of swine holdings and their farmers.

Step 2: Publications investigating all three compartments
Inclusion of publications of the folder "Inclusion_def" providing information about all 3 compartments (human, animal, environment) and sorting them into following categories:

- "3 criteria exclusion": Publication does not provide information in all three dimensions (animal-human-environment).
- "3 criteria inclusion": Publication provides information in all three dimensions/compartments

- “3 criteria inclusion evtl”: Publication provides information in all three dimensions/compartments in a broader sense. E.g.: Sampling of sewage with different anthropogenic influence or influence of farm environment, food as environmental component (e.g. salad), direct farm environment as environment (e.g. swabs of livestock buildings)
- “3 criteria inclusion wildlife”: Publication provides information in two of the three dimensions (animal and human or environment and animal) and additionally in wildlife (e.g. mussels, oysters, fish, wild gorillas)

Comment: Since there were so many publications in the folder “inclusion_def” we decided to process only these selected publications and leave the folder “inclusion_evtl” apart.

Step 3: Finding consensus
Finding Consensus.

- “3 criteria inclusion consensus”: Publications providing information in all three dimensons (animal-human-environment) included either by Keira Spinner and Hélène Meier or by only one of both and included finding consensus.
- “3 criteria inclusion consensus review”: Publications that are in another format (e.g. reviews, letter to editor) and relevant for our systematic review. Only Keira Spinners reviews are included in here because Hélène Meier sorted them out in Step 1.

Step 4: Correction of consensus
After finding consensus this analysis plan was written. While reading the papers in the folder “3 criteria inclusion consensus” we noticed that some papers didn’t fit our inclusion criteria well. So we had to correct and overthink our “3 criteria inclusion consensus” folder again and create the folder “3 criteria inclusion consensus korr”. The following paper were excluded:

- Acar, S., et al. (2017). “Phenotyping and genetic characterization of Salmonella enterica isolates from Turkey revealing arise of different features specific to geography.” Int J Food Microbiol 241: 98-107.
- Alonso, C. A., et al. (2017). "Analysis of blaSHV-12-carrying Escherichia coli clones and plasmids from human, animal and food sources." J Antimicrob Chemother **72**(6): 1589-1596.
- Aslantas, O., et al. (2012). “Isolation and molecular characterization of methicillin-resistant staphylococci from horses, personnel and environmental sites at an equine hospital in Turkey.” J Vet Med Sci **74**(12): 1583-1588.
- Couto, N., et al. (2015). “Clonal diversity, virulence patterns and antimicrobial and biocide susceptibility among human, animal and environmental MRSA in Portugal.” J Antimicrob Chemother **70**(9): 2483-2487.
- Deng, Y., et al. (2011). "Dissemination of IncFII plasmids carrying rmtB and qepA in Escherichia coli from pigs, farm workers and the environment." Clin Microbiol Infect **17**(11): 1740-1745.
- Phumthanakorn, N., et al. (2018). “Enterotoxin gene profile of methicillin-resistant Staphylococcus pseudintermedius isolates from dogs, humans and the environment.” J Med Microbiol **67**(6): 866-873.
- Rousham, E., et al. (2018). “Spatial and temporal variation in the community prevalence of antibiotic resistance in Bangladesh: an integrated surveillance study protocol.” BMJ Open **8**(4): e023158.
- Stalsby Lundborg, C., et al. (2015). “Protocol: a ‘One health’ two year follow-up, mixed methods study on antibiotic resistance, focusing children under 5 and their environment in rural India.” BMC Public Health **15**: 1321.

- Stedtfeld, R. D., et al. (2016). “Antimicrobial resistance dashboard application for mapping environmental occurrence and resistant pathogens.” FEMS Microbiol Ecol **92**(3).
- Sun, Q., et al. (2018). “Study protocol for One Health data collections, analyses and intervention of the Sino-Swedish integrated multisectoral partnership for antibiotic resistance containment (IMPACT).” BMJ Open **8**(1): e017832.
- Zambri, M., et al. (2019). “Novel virulence, antibiotic resistance and toxin gene-specific PCR-based assays for rapid pathogenicity assessment of Arcobacter faecis and Arcobacter lanthieri.” BMC Microbiol **19**(1): 11.

For more detailed information concerning reasons for exclusion of these papers see Table S5.

C) Numbers of sorted publications
Full search: 9115 results

Step 1: Publications investigating two out of three compartments
In Step 1 (without finding consensus) distribution was as followed:

Folder name	Number of Publications Keira Spinner	Number of Publications Hélène Meier
“Access not working”	173	173
“Duplicates”	3	3
“Exclusion_def”	8202	6067
“Inclusion_def”	647	887
“Inclusion_evtl”	-	1074
“øLanguage”	233	156
“øLanguage but important”	32	16
“OtherFormatNotRelevant”	-	233
“Retracted article”	1	1
“TextsforSources/otherformat”	-	521

Table S1. *Distribution of papers in EndNote library in step 1.*

Step 2: Publications investigating all three compartments
In Step 2 (without finding consensus) distribution was as followed:

Folder name	Number of Publications Keira Spinner	Number of Publications Hélène Meier
“Access not working”	173	173
“Duplicates”	3	3
“Exclusion_def”	8202	6067
“Inclusion_def”	647	887
“Inclusion_evtl”	-	1074
“øLanguage”	233	156
“øLanguage but important”	32	16
“OtherFormatNotRelevant”	-	233
“Retracted article”	1	1
“TextsforSources/otherformat”	-	521
“3 criteria exclusion”	547	679

“3 criteria inclusion”	132	110
“3 criteria inclusion evtl”	-	78
“3 criteria inclusion wildlife”	-	20

Table S2. *Distribution of papers in EneNote library in step 2.*

Step 3: Finding consensus
In Step 3 after finding consensus distribution was as followed:

Folder name	Number of Publications Keira Spinner	Number of Publications Hélène Meier
“Access not working”	173	173
“Duplicates”	3	3
“Exclusion_def”	8202	6067
“Inclusion_def”	647	887
“Inclusion_evtl”	-	1074
“øLanguage”	233	156
“øLanguage but important”	32	16
“OtherFormatNotRelevant”	-	233
“Retracted article”	1	1
“TextsforSources/otherformat”	-	521
“3 criteria exclusion”	547	679
“3 criteria inclusion”	132	110
“3 criteria inclusion consensus”	100	100
“3 criteria inclusion consensus review”	50	50
“3 criteria inclusion evtl”	-	78
“3 criteria inclusion wildlife”	-	20

Table S3. *Distribution of papers in EndNote library in step 3.*

Step 4: Correction of consensus
In Step 4 corrections were done while reading the papers for the analysis plan:

Folder name	Number of Publications Keira Spinner	Number of Publications Hélène Meier
“Access not working”	173	173
“Duplicates”	3	3
“Exclusion_def”	8202	6067
“Inclusion_def”	647	887
“Inclusion_evtl”	-	1074
“øLanguage”	233	156
“øLanguage but important”	32	16
“OtherFormatNotRelevant”	-	233
“Retracted article”	1	1
“TextsforSources/otherformat”	-	521
“3 criteria exclusion”	574	679
“3 criteria inclusion”	132	110
“3 criteria inclusion consensus”	100	100

“3 criteria inclusion consensus review”	50	50
“3 criteria inclusion consensus korr”	89	89
“3 criteria inclusion evtl”	-	78
“3 criteria inclusion wildlife”	-	20

Table S4. *Distribution of papers in EndNote library step 4.*

Exclusions after analysis of observed compartments (environment, animal, human) (Step 4)
While doing a first literature analysis after finding consensus, further studies were excluded for the following reasons:

Paper excluded in step 4	Main reason for exclusion	Additional information
Acar, S., et al. (2017). "Phenotyping and genetic characterization of Salmonella enterica isolates from Turkey revealing arise of different features specific to geography." Int J Food Microbiol 241: 98-107.	Not all three compartments represented.	Environmental compartment is lacking, because no *Salmonella enterica* strains could be isolated from environmental samples (pistachios, pepper, paprika)
Alonso, C. A., et al. (2017). "Analysis of blaSHV-12-carrying Escherichia coli clones and plasmids from human, animal and food sources." J Antimicrob Chemother **72**(6): 1589-1596.	Not all three compartments represented.	Environmental compartment is lacking (but sampling of wildlife (i.e. wild birds)).
Aslantas, O., et al. (2012). "Isolation and molecular characterization of methicillin-resistant staphylococci from horses, personnel and environmental sites at an equine hospital in Turkey." J Vet Med Sci **74**(12): 1583-1588.	Investigating all three compartments, but compartments being closely related to each other.	Only direct environment of an equine hospital is being sampled. Papers investigating all three compartments where the environmental sector is closely related to the animal and human sector were excluded.
Couto, N., et al. (2015). "Clonal diversity, virulence patterns and antimicrobial and biocide susceptibility among human, animal and environmental MRSA in Portugal." J Antimicrob Chemother **70**(9): 2483-2487.	Investigating all three compartments, but compartments being closely related to each other.	Only direct environment of pigs (environmental dust samples from breeding pig sheds) were investigated as environmental component. Papers investigating all three compartments where the environmental sector is closely related to the animal and human sector were excluded.
Deng, Y., et al. (2011). "Dissemination of IncFII plasmids carrying rmtB and qepA in Escherichia coli from pigs, farm workers and the environment." Clin Microbiol Infect **17**(11): 1740-1745.	Investigating all three compartments, but compartments being closely related to each other.	All samples originiated from one farm and from the direct environment of animals.<br>Papers investigating all three compartments where the environmental sector is closely related to the animal and human sector were excluded.<br>But well documentation of clonal relatedness between isolates.

Phumthanakorn, N., et al. (2018). "Enterotoxin gene profile of methicillin-resistant Staphylococcus pseudintermedius isolates from dogs, humans and the environment." J Med Microbiol **67**(6): 866-873.	Not investigating antibiotic resistance.<br><br>Investigating all three compartments, but compartments being closely related to each other.	Study is determining enterotoxin gene (virulence gene) profiles of methicillin-resistant Staph. pseudintermedius, but does not investigate antibiotic resistance profiles. Methicillin resistance was an inclusion criteria for the isolates investigated in the study, but otherwise this study has nothing to do with antimicrobial resistance. Additionally samples were only taken from dogs at a Veterinary Teaching Hospital, their owners/veterinarians and the direct environment of the Veterinary Hospital (door handles, etc.).
Rousham, E., et al. (2018). "Spatial and temporal variation in the community prevalence of antibiotic resistance in Bangladesh: an integrated surveillance study protocol." BMJ Open **8**(4): e023158.	Other format (study protocol).	Article is describing and planning an integrated OneHealth study on antibiotic resistance (study protocol).<br>It was a common decision to only include papers generating (primary) data and investigating isolates for their resistance patterns. Theoretical scenarios (e.g. epidemiological modeling) or other formats (e.g. study protocols, reviews, reports) were excluded.
Stalsby Lundborg, C., et al. (2015). "Protocol: a 'One health' two year follow-up, mixed methods study on antibiotic resistance, focusing children under 5 and their environment in rural India." BMC Public Health **15**: 1321.	Other format (study protocol).	Article is describing and planning an integrated OneHealth study on antibiotic resistance (study protocol).<br>It was a common decision to only include papers generating (primary) data and investigating isolates for their resistance patterns. Theoretical scenarios (e.g. epidemiological modeling) or other formats (e.g. study protocols, reviews, reports) were excluded.?
Stedtfeld, R. D., et al. (2016). "Antimicrobial resistance dashboard application for mapping environmental occurrence and resistant pathogens." FEMS Microbiol Ecol **92**(3).	Other format.<br><br>Not all three compartments represented.	Other format: Study describes the establishment of a dashboard application for antimicrobial resistance, using some samples (clinical and environmental) to present it using examples.<br>Additionally the animal compartment is lacking.
Sun, Q., et al. (2018). "Study protocol for One Health data collections, analyses and intervention of the Sino-Swedish integrated multisectoral partnership for antibiotic resistance containment (IMPACT)." BMJ Open **8**(1): e017832.	Other format (study protocol).	Article is describing and planning an integrated OneHealth study on antibiotic resistance (study protocol).<br>It was a common decision to only include papers generating (primary) data and investigating isolates for their resistance patterns. Theoretical scenarios (e.g. epidemiological modeling) or other formats (e.g. study protocols, reviews, reports) were excluded.
Zambri, M., et al. (2019). "Novel virulence, antibiotic resistance and toxin gene-specific PCR-	Not focusing on investigation of	Focus of the article is on establishing a new method for detection of two specific ARGs using PCR assays. This

based assays for rapid pathogenicity assessment of Arcobacter faecis and Arcobacter lanthieri." BMC Microbiol **19**(1): 11.	antimicrobial resistance.	assay is being tested with different isolates of the three compartments, but the study focus is not on AMR.

Table S5. *Reasons for correction of consensus and exclusion of papers in step 4.*

D) Papers included in Systematic Review

1. Abreo, E. and N. Altier (2019). “Pangenome of Serratia marcescens strains from nosocomial and environmental origins reveals different populations and the links between them.” Sci Rep **9**(1): 46
2. Adesiji, Y. O., et al. (2014). "Antimicrobial-resistant genes associated with Salmonella spp. isolated from human, poultry, and seafood sources." Food Sci Nutr **2**(4): 436-442.
3. Ajiboye, R. M., et al. (2009). "Global spread of mobile antimicrobial drug resistance determinants in human and animal Escherichia coli and Salmonella strains causing community-acquired infections." Clin Infect Dis **49**(3): 365-371.
4. Al-Bahry, S. N., et al. (2007). "Antibiotic-resistant Salmonella spp. from human and non-human sources in Oman." East Mediterr Health J **13**(1): 49-55.
5. Alcaide, E., et al. (2010). "Mechanisms of quinolone resistance in Aeromonas species isolated from humans, water and eels." Res Microbiol **161**(1): 40-45.
6. Antunes, P., et al. (2006). "Characterization of antimicrobial resistance and class 1 and 2 integrons in Salmonella enterica isolates from different sources in Portugal." J Antimicrob Chemother **58**(2): 297-304.
7. Atterby, C., et al. (2017). "ESBL-producing Escherichia coli in Swedish gulls-A case of environmental pollution from humans?" PLoS One **12**(12): e0190380.
8. Bertsch, D., et al. (2014). "Antimicrobial susceptibility and antibiotic resistance gene transfer analysis of foodborne, clinical, and environmental Listeria spp. isolates including Listeria monocytogenes." Microbiologyopen **3**(1): 118-127.
9. Bonke, R., et al. (2011). "Antimicrobial susceptibility and distribution of beta-lactamase A (blaA) and beta-lactamase B (blaB) genes in enteropathogenic Yersinia species." Microb Drug Resist **17**(4): 575-581.
10. Boonyasiri, A., et al. (2014). "Prevalence of antibiotic resistant bacteria in healthy adults, foods, food animals, and the environment in selected areas in Thailand." Pathog Glob Health **108**(5): 235-245.
11. Busani, L., et al. (2004). "Antibiotic resistance in Salmonella enterica serotypes Typhimurium, Enteritidis and Infantis from human infections, foodstuffs and farm animals in Italy." Epidemiol Infect **132**(2): 245-251.
12. Cao, Y., et al. (2016). "Prevalence and characterization of resistance genes with genetic clones among Staphylococcus aureus isolates obtained from various sources in China." J Med Microbiol **65**(6): 569-571.
13. Chao, G., et al. (2013). "Phenotypic and genotypic characterization of methicillin-resistant Staphylococcus aureus (MRSA) and methicillin-susceptible Staphylococcus aureus (MSSA) from different sources in China." Foodborne Pathog Dis **10**(3): 214-221.
14. Chen, K., et al. (2017). "Widespread distribution of mcr-1-bearing bacteria in the ecosystem, 2015 to 2016." Euro Surveill **22**(39).
15. Chen, X., et al. (2017). "Detection and dissemination of the colistin resistance gene, mcr-1, from isolates and faecal samples in China." J Med Microbiol **66**(2): 119-125
16. Davis, J. A. and C. R. Jackson (2009). "Comparative antimicrobial susceptibility of Listeria monocytogenes, L. innocua, and L. welshimeri." Microb Drug Resist **15**(1): 27-32.
17. Dawes, F. E., et al. (2010). "Distribution of class 1 integrons with IS26-mediated deletions in their 3'-conserved segments in Escherichia coli of human and animal origin." PLoS One **5**(9): e12754.
18. Deredjian, A., et al. (2011). "Antibiotic and metal resistance among hospital and outdoor strains of Pseudomonas aeruginosa." Res Microbiol **162**(7): 689-700.
19. Dhaka, P., et al. (2016). "Genetic diversity and antibiogram profile of diarrhoeagenic Escherichia coli pathotypes isolated from human, animal, foods and associated environmental sources." Infect Ecol Epidemiol **6**: 31055.
20. Dionisi, A. M., et al. (2011). "Molecular characterisation of multidrug-resistant Salmonella enterica serotype Infantis from humans, animals and the environment in Italy." Int J Antimicrob Agents **38**(5): 384-389.
21. Esteve, C., et al. (2012). "Aeromonas hydrophila subsp. dhakensis isolated from feces, water and fish in Mediterranean Spain." Microbes Environ **27**(4): 367-373.
22. Esteve, C., et al. (2015). "Multidrug-resistant (MDR) Aeromonas recovered from the metropolitan area of Valencia (Spain): diseases spectrum and prevalence in the environment." Eur J Clin Microbiol Infect Dis **34**(1): 137-145.
23. Fernandes, M. R., et al. (2016). "Silent dissemination of colistin-resistant Escherichia coli in South America could contribute to the global spread of the mcr-1 gene." Euro Surveill **21**(17).
24. Fernandes, S. A., et al. (2017). "Prevalence of Extended-Spectrum beta-Lactamases CTX-M-8 and CTX-M-2-Producing Salmonella Serotypes from Clinical and Nonhuman Isolates in Brazil." Microb Drug Resist **23**(5): 580-589.
25. Field, W. and R. Hershberg (2015). "Alarmingly High Segregation Frequencies of Quinolone Resistance Alleles within Human and Animal Microbiomes Are Not Explained by Direct Clinical Antibiotic Exposure." Genome Biol Evol **7**(6): 1743-1757.

26. Fitzpatrick, D. and F. Walsh (2016). "Antibiotic resistance genes across a wide variety of metagenomes." FEMS Microbiol Ecol **92**(2).
27. Frazao, M. R., et al. (2017). "Antimicrobial resistance and plasmid replicons in Yersinia enterocolitica strains isolated in Brazil in 30 years." Braz J Infect Dis **21**(4): 477-480.
28. Freitas, A. R., et al. (2017). "Detection of optrA in the African continent (Tunisia) within a mosaic Enterococcus faecalis plasmid from urban wastewaters." J Antimicrob Chemother **72**(12): 3245-3251.
29. Freitas, A. R., et al. (2010). "Global spread of the hyl(Efm) colonization-virulence gene in megaplasmids of the Enterococcus faecium CC17 polyclonal subcluster." Antimicrob Agents Chemother **54**(6): 2660-2665.
30. Gallati, C., et al. (2013). "Characterization of Salmonella enterica subsp. enterica serovar 4,[5],12:i:- clones isolated from human and other sources in Switzerland between 2007 and 2011." Foodborne Pathog Dis **10**(6): 549-554.
31. Gamboa-Coronado Mdel, M., et al. (2011). "[Molecular characterization and antimicrobial resistance of Clostridium perfringens isolates of different origins from Costa Rica]." Rev Biol Trop **59**(4): 1479-1485.
32. Gatica, J., et al. (2019). "Comparative Metagenomics and Network Analyses Provide Novel Insights Into the Scope and Distribution of beta-Lactamase Homologs in the Environment." Front Microbiol **10**: 146.
33. Gonzalez, M., et al. (2009). "[Antimicrobial susceptibility and molecular typing of Enterococcus faecium isolated from humans, chickens and environment in Canary Islands (Spain)]." Rev Esp Quimioter **22**(3): 120-126.
34. Gonzalez-Rey, C., et al. (2004). "Serotypes and anti-microbial susceptibility of Plesiomonas shigelloides isolates from humans, animals and aquatic environments in different countries." Comp Immunol Microbiol Infect Dis **27**(2): 129-139.
35. Hauschild, T., et al. (2007). "Aminoglycoside resistance in members of the Staphylococcus sciuri group." Microb Drug Resist **13**(2): 77-84.
36. Hu, J., et al. (2008). "Phenotyping and genotyping of antibiotic-resistant Escherichia coli isolated from a natural river basin." Environ Sci Technol **42**(9): 3415-3420.
37. Hu, Y. Y., et al. (2013). "Molecular typing of CTX-M-producing escherichia coli isolates from environmental water, swine feces, specimens from healthy humans, and human patients." Appl Environ Microbiol **79**(19): 5988-5996.
38. Hayford, A. E., et al. (2015). "Genetic and resistance phenotypic subtyping of Salmonella Saintpaul isolates from various food sources and humans: Phylogenetic concordance in combinatory analyses." Infect Genet Evol **36**: 92-107.
39. Heider, L. C., et al. (2009). "Genetic and phenotypic characterization of the bla(CMY) gene from Escherichia coli and Salmonella enterica isolated from food-producing animals, humans, the environment, and retail meat." Foodborne Pathog Dis **6**(10): 1235-1240.
40. Ibekwe, A. M., et al. (2011). "Genetic diversity and antimicrobial resistance of Escherichia coli from human and animal sources uncovers multiple resistances from human sources." PLoS One **6**(6): e20819.
41. Janam, R., et al. (2011). "Antibiogram and genotyping of Pseudomonas aeruginosa isolated from human, animal, plant, water and soil sources in north India." Southeast Asian J Trop Med Public Health **42**(6): 1477-1488.
42. Keelara, S., et al. (2014). "Comparative phenotypic and genotypic characterization of temporally related nontyphoidal Salmonella isolated from human clinical cases, pigs, and the environment in North Carolina." Foodborne Pathog Dis **11**(2): 156-164.
43. Keelara, S. and S. Thakur (2014). "Dissemination of plasmid-encoded AmpC beta-lactamases in antimicrobial resistant Salmonella serotypes originating from humans, pigs and the swine environment." Vet Microbiol **173**(1-2): 76-83.
44. Kyselkova, M., et al. (2012). "Tetracycline resistance and presence of tetracycline resistance determinants tet(V) and tap in rapidly growing mycobacteria from agricultural soils and clinical isolates." Microbes Environ **27**(4): 413-422.
45. Kim, D. W., et al. (2018). "Revisiting Polymorphic Diversity of Aminoglycoside N-Acetyltransferase AAC(6')-Ib Based on Bacterial Genomes of Human, Animal, and Environmental Origins." Front Microbiol **9**: 1831.
46. Knetsch, C. W., et al. (2018). "Zoonotic Transfer of Clostridium difficile Harboring Antimicrobial Resistance between Farm Animals and Humans." J Clin Microbiol **56**(3).
47. Kuang, D., et al. (2018). "Increase in Ceftriaxone Resistance and Widespread Extended-Spectrum beta-Lactamases Genes Among Salmonella enterica from Human and Nonhuman Sources." Foodborne Pathog Dis **15**(12): 770-775
48. Kuhn, I., et al. (2005). "Occurrence and relatedness of vancomycin-resistant enterococci in animals, humans, and the environment in different European regions." Appl Environ Microbiol **71**(9): 5383-5390.
49. Lau, C. H., et al. (2017). "NOVEL ANTIBIOTIC RESISTANCE DETERMINANTS FROM AGRICULTURAL SOIL EXPOSED TO ANTIBIOTICS WIDELY USED IN HUMAN MEDICINE AND ANIMAL FARMING." Appl Environ Microbiol.

50. Lindmark, H., et al. (2004). "Genetic characterization and antibiotic resistance of Campylobacter jejuni isolated from meats, water, and humans in Sweden." J Clin Microbiol **42**(2): 700-706.
51. Lupindu, A. M., et al. (2015). "Transmission of antibiotic-resistant Escherichia coli between cattle, humans and the environment in peri-urban livestock keeping communities in Morogoro, Tanzania." Prev Vet Med **118**(4): 477-482.
52. Ma, L., et al. (2016). "Metagenomic Assembly Reveals Hosts of Antibiotic Resistance Genes and the Shared Resistome in Pig, Chicken, and Human Feces." Environ Sci Technol **50**(1): 420-427.
53. Ma, Y., et al. (2018). "High-levels of resistance to quinolone and cephalosporin antibiotics in MDR-ACSSuT Salmonella enterica serovar Enteritidis mainly isolated from patients and foods in Shanghai, China." Int J Food Microbiol **286**: 190-196.
54. Montealegre, M. C., et al. (2018). "Risk Factors for Detection, Survival, and Growth of Antibiotic-Resistant and Pathogenic Escherichia coli in Household Soils in Rural Bangladesh." Appl Environ Microbiol **84**(24).
55. Mourao, J., et al. (2014). "Characterization of the emerging clinically-relevant multidrug-resistant Salmonella enterica serotype 4,[5],12:i:- (monophasic variant of S. Typhimurium) clones." Eur J Clin Microbiol Infect Dis **33**(12): 2249-2257. –
56. Mutters, N. T., et al. (2016). "Comparison of livestock-associated and health care-associated MRSA-genes, virulence, and resistance." Diagn Microbiol Infect Dis **86**(4): 417-421.
57. Nesme, J., et al. (2014). "Large-scale metagenomic-based study of antibiotic resistance in the environment." Curr Biol **24**(10): 1096-1100.
58. .Nogrady, N., et al. (2010). "Molecular and pathogenic characterization of Salmonella enterica serovar Bovismorbificans strains of animal, environmental, food, and human origin in Hungary." Foodborne Pathog Dis **7**(5): 507-513.
59. Noll, M., et al. (2018). "Antibiotic susceptibility of 259 Listeria monocytogenes strains isolated from food, food-processing plants and human samples in Germany." J Infect Public Health **11**(4): 572-577.
60. Novais, C., et al. (2008). "Diversity of Tn1546 and its role in the dissemination of vancomycin-resistant enterococci in Portugal." Antimicrob Agents Chemother **52**(3): 1001-1008.
61. Ottaviani, D., et al. (2018). "Molecular characterization and drug susceptibility of non-O1/O139 V. cholerae strains of seafood, environmental and clinical origin, Italy." Food Microbiol **72**: 82-88.
62. Ojer-Usoz, E., et al. (2017). "Clonal Diversity of ESBL-Producing Escherichia coli Isolated from Environmental, Human and Food Samples." Int J Environ Res Public Health **14**(7).
63. Oravcova, V., et al. (2017). "Vancomycin-resistant enterococci with vanA gene in treated municipal wastewater and their association with human hospital strains." Sci Total Environ **609**: 633-643.
64. Oravcova, V., et al. (2018). "Wild corvid birds colonized with vancomycin-resistant Enterococcus faecium of human origin harbor epidemic vanA plasmids." Environ Int **118**: 125-133.
65. Pehrsson, E. C., et al. (2016). "Interconnected microbiomes and resistomes in low-income human habitats." Nature **533**(7602): 212-216.
66. Poppe, C., et al. (2006). "Characterization of antimicrobial resistance of Salmonella Newport isolated from animals, the environment, and animal food products in Canada." Can J Vet Res **70**(2): 105-114.
67. Pailhories, H., et al. (2015). "Diversity of Acinetobacter baumannii strains isolated in humans, companion animals, and the environment in Reunion Island: an exploratory study." Int J Infect Dis **37**: 64-69.
68. Pal, C., et al. (2016). "The structure and diversity of human, animal and environmental resistomes." Microbiome **4**(1): 54.
69. Park, M., et al. (2010). "Phenotypic and genotypic characterization of tetracycline and minocycline resistance in Clostridium perfringens." Arch Microbiol **192**(10): 803-810.
70. Purohit, M. R., et al. (2017). "Antibiotic Resistance in an Indian Rural Community: A 'One-Health' Observational Study on Commensal Coliform from Humans, Animals, and Water." Int J Environ Res Public Health **14**(4).
71. Rodriguez, I., et al. (2009). "Extended-spectrum {beta}-lactamases and AmpC {beta}-lactamases in ceftiofur-resistant Salmonella enterica isolates from food and livestock obtained in Germany during 2003-07." J Antimicrob Chemother **64**(2): 301-309.
72. Rozynek, E., et al. (2010). "Genetic similarity of Campylobacter isolates in humans, food, and water sources in central Poland." Foodborne Pathog Dis **7**(5): 597-600.
73. Sahoo, K. C., et al. (2012). "Geographical variation in antibiotic-resistant Escherichia coli isolates from stool, cow-dung and drinking water." Int J Environ Res Public Health **9**(3): 746-759.
74. Savic, D., et al. (2016). "Antimicrobial susceptibility and beta-lactamase production in Bacillus cereus isolates from stool of patients, food and environment samples." Vojnosanit Pregl **73**(10): 904-909.
75. Seixas, R., et al. (2016). "Phenotypic and Molecular Characterization of Salmonella 1,4,[5],12:i:- R-Type ASSuT Isolates from Humans, Animals, and Environment in Portugal, 2006-2011." Foodborne Pathog Dis **13**(11): 633-641.
76. Sianglum, W., et al. (2007). "Analysis of gyrA mutations related to quinolone resistance in Escherichia coli isolates originating from pet, human, vegetable and ice in Bangkok and vicinity." Southeast Asian J Trop Med Public Health **38**(6): 1095-1101.

77. Silveira, E., et al. (2014). "Co-transfer of resistance to high concentrations of copper and first-line antibiotics among Enterococcus from different origins (humans, animals, the environment and foods) and clonal lineages." J Antimicrob Chemother **69**(4): 899-906.
78. Soni, D. K., et al. (2013). "Characterization of Listeria monocytogenes isolated from Ganges water, human clinical and milk samples at Varanasi, India." Infect Genet Evol **14**: 83-91.
79. Thong, K. L. and C. P. Ang (2011). "Genotypic and phenotypic differentiation of Salmonella enterica serovar Paratyphi B in Malaysia." Southeast Asian J Trop Med Public Health **42**(5): 1178-1189.
80. Veldman, K., et al. (2011). "International collaborative study on the occurrence of plasmid-mediated quinolone resistance in Salmonella enterica and Escherichia coli isolated from animals, humans, food and the environment in 13 European countries." J Antimicrob Chemother **66**(6): 1278-1286.
81. Versluis, D., et al. (2015). "Mining microbial metatranscriptomes for expression of antibiotic resistance genes under natural conditions." Sci Rep **5**: 11981.
82. Yang, X., et al. (2015). "Antimicrobial Resistance and Molecular Typing of Salmonella Stanley Isolated from Humans, Foods, and Environment." Foodborne Pathog Dis **12**(12): 945-949.
83. Zeng, J., et al. (2019). "Metagenomic insights into the distribution of antibiotic resistome between the gut-associated environments and the pristine environments." Environ Int **126**: 346-354.
84. Zahid, S., et al. (2017). "Prevalence and genetic profiling of tetracycline resistance (Tet-R) genes and transposable element (Tn916) in environmental Enterococcus species." Microb Pathog **111**: 252-261.
85. Zhang, S. H., et al. (2015). "Prevalence of antibiotic resistance genes in antibiotic-resistant Escherichia coli isolates in surface water of Taihu Lake Basin, China." Environ Sci Pollut Res Int **22**(15): 11412-11421.
86. Zhang, W., et al. (2014). "Phylogeny and phenotypes of clinical and environmental Shiga toxin-producing Escherichia coli O174." Environ Microbiol **16**(4): 963-976.
87. Zhang, Z., et al. (2018). "Comparative Study on Antibiotic Resistance and DNA Profiles of Salmonella enterica Serovar Typhimurium Isolated from Humans, Retail Foods, and the Environment in Shanghai, China." Foodborne Pathog Dis **15**(8): 481-488.
88. Zhao, J., et al. (2010). "Prevalence and dissemination of oqxAB in Escherichia coli isolates from animals, farmworkers, and the environment." Antimicrob Agents Chemother **54**(10): 4219-4224.
89. Zurfluh, K., et al. (2015). "Replicon typing of plasmids carrying blaCTX-M-15 among Enterobacteriaceae isolated at the environment, livestock and human interface." Sci Total Environ **521-522**: 75-78.

2. Hermeneutic literature analysis

Analysis of literature included into the systematic review was performed using Atlas.ti 8, a scientific software for hermeneutic analysis of literature.

Established Code Groups and Codes

In total 167 Codes were established and subdivided into 14 Code Groups.

Code Group	Code	Description of Code
definition/ explanation	definition_information – AMR patterns	Study mentions the definition of certain AMR patterns (e.g. ASSuT = resistance to ampicillin, streptomycin, sulfonamides and tetracycline in monophasic variant of Salmonella typhimurium).
	definition_information - ESBL	What are ESBL?
	definition_information – ways of acquisition of AMR by bacteria	How can bacteria acquire resistance to antibiotics? Study explains the different ways how AMR can spread or occurr de novo using knowledge from studies done before.
	definition_methods - conjugation experiments	Study explains the purpose of conjugation experiments (concerning plasmids).
	definition_methods - explanation of MLST	Study explaining Multilocus Sequence Typing as an important tool for tracing of gene transmission and analysis of pathogen evolution (source tracking).
	definition_methods - explanation of WGS	definition of e.g. frequent used methods
	definition_methods - explanation of whole genome multilocus sequence typing	definition of e.g. frequent used methods
	definition_methods - methods for phylogeny/source tracking	Study provides information concerning (different) methods for phylogeny/source tracking (genetic relatedness of bacteria).
	definition_methods - PFGE	Study explains some features of pulsed-field gel electrophoresis (e.g. that it is the gold standard test for detection of genotypic diversity).
	definition_methods - southern blot	Authors describe Southern Blot analysis/Southern hybridization (use, aims, ...).
	definition_mobile genetic element - Tn1546	Providing information about transposon Tn1546.
	definition_mobile genetic elements - integrons	Definition of “integrons” mentioned in study.
	definition_mobile genetic elements - plasmids	Study provides information concerning plasmids (e.g. plasmids belonging to IncH family and their characteristics).
	definitions_information - antibiotic resistance gene determinants	Definition of the term “antibiotic resistance gene determinants” (ARGD).
study aims	aims - general	general aims of the study

	aims – source tracking/phylogeny	aims of study, e.g. source tracking of bacteria and antimicrobial resistance genes
information	information - (probable) reasons for spread of/factors leading to AMR	Study provides information (already existing from previous studies) about (probable) causes for spread of AMR (e.g. extensive use of ABC in hospital settings). And factors (e.g. coselection with metal resistance) leading to AMR/selection of AMR bacteria.<br>*Code newly established at Derdjian (2011).*
	information - AMR between sectors	Study provides information concerning antimicrobial resistance between different sectors (e.g. wild birds as frequent carriers of ESBL-producing E. coli strains with genotypic characteristics similar to strains found in humans)
	information - antibiotic agents	Study provides information about antibiotic agents (e.g. indications, known resistances, etc).
	information - ARGs	Study provides information known so far (from studies done before) concerning ARGs (e.g. their distribution, presence, mechanisms...).
	information - bacteria	Study provides information concerning the bacteria itself (e.g. its pathogenicity for a certain sector (e.g. which serotype is most commonly involved in human infection) or association of a certain serotype with a certain sector).
	information - classification of bacteria	Study provides information concerning classification of bacteria investigated in study (e.g. biotypes and which of them are associated with human infection).
	information - comparison of methods (phylogeny/source tracking)	Authors discuss different methods for epidemiologic characterization of bacteria (e.g. what are advantages of techniques?).<br>*Code newly established at Hayford (2015).*
	information - coselection of AMR	Study provides information (already known before from previous studies) concerning factors leading to coselection of AMR (e.g. metal resistance leading to AMR).<br>*Code newly established at Deredjian (2011).*
	information - disease caused by bacteria	Study provides information concerning disease/impacts caused by the bacteria species being investigated.<br>*Code newly established at Dionisi (2011). Before those information were either not coded at all or in the code "information - bacteria".*
	information - genotypic resistance mechanisms	Study providing information about genotypic resistance mechanisms (e.g. resistance mechanisms leading to

		resistance to quinolones such as gyrase or topoisomerase mutations).
	information - inconsistent results between studies	Study mentions that different results were achieved between studies concerning antimicrobial resistance.
	information - interesting inputs/thoughts	Study provides interesting inputs/thoughts concerning AMR (e.g. role of non-pathogenic bacteria as reservoirs of ARGs that can be transferred to related, pathogenic bacteria).<br>*Code newly established at Davis (2009).*
	information - intrinsic AMR of bacteria	Study provides information about already known intrinsic/innate antimicrobial resistance (mechanism) to ABC.<br>*Code newly established at Deredjian (2011).*
	information - lack of knowledge concerning antibiotic resistance	Study provides information about knowledge gap concerning antibiotic resistance (e.g. in a certain region of the world on a certain bacteria species).
	information - measures for reduction/control of AMR	Study provides information concerning measures that were already implicated to reduce AMR (e.g. ban of ABC as food additives).
	information - methods/bacteria used for detection of AMR	Study provides information on certain special methods/ bacteria used for detection of antimicrobial resistance (e.g. E. coli that is often used as a sentinel for monitoring AMR in faecal bacteria).
	information - MRSA	Study provides information concerning methicillin-resistant Staph. aureus (e.g. genes carried by hospital-associated vs. community-associated MRSA/methicillin-susceptible S. aureus).
	information - prevalence/presence of antibiotic resistant bacteria	Study provides information (taken from other sources) about prevalence/presence of antimicrobial resistant bacteria (e.g. Salmonella). E.g. distribution of prevalence of antimicrobial resistant bacteria between sectors.
	information - spread of antimicrobial resistance	Study providing information (not part of study) concerning spread/transmission of antimicrobial resistance between sectors (e.g. human and animal) - either referring to studies done before or as a statement.
	information - spread of bacteria between sectors	Study provides information on spread of specific bacteria between sectors (e.g. 99% of listeriosis cases in humans are due to food-borne transmission of the bacteria).<br>Information might be helpful sometimes (especially for pathogens as Listeria spp. not belongig to a healthy human microbiota) to elucidate transmission of antimicrobial resistant bacteria indirectly. (e.g. if there is a listeriosis patient with AMR listeria it is very likely coming from food - and therefore AMR might have arised in the food sector? Although

		selection of AMR pathogens could also have occurred in the diseased patient himself).
	information - spread of mobile genetic elements	Study providing information/hypothesising about spread of mobile genetic elements (e.g. from other studies done before).
	introduction HM - impact of AMR	Study providing information that could be useful for introduction/discussion/conclusion part of hemeier review.
	introduction HM – mechanisms of acquisition of AMR	How can bacteria acquire resistance to antibiotics? Study explains the different ways how AMR can spread or occurr de novo using knowledge from studies done before.
	open question/information – mobile genetic elements	Study providing information (e.g. results from previous studies) or mentioning open questions concerning mobile genetic elements.
	open question/information – supporting One Health approach	Study mentioning and supporting OneHealth approach in terms of solving problem of antimicrobial resistance.
methods	methods – (whole) genome sequencing approach	Metagenomic study using (whole) genome sequencing looking for AMR.
	methods - ABCs used for antimicrobial susceptibility testing (phenotypic)	Which antimicrobials were used for antimicrobial susceptibility testing? (e.g. ampicillin, etc.)
	methods - additional susceptibility testing (phenotypic)	Which additional susceptibility testing methods (e.g. double disc synergy test for detection of ESBL production/ expression of ESBL genes) were performed? Maybe even how (e.g. using which discs)?
	methods - analysis of ARGs	What methods were applied in the present study for analysis of antibiotic resistance genes? (e.g. PCR assays for amplification of quinolone resistance determining regions of certain ARGs followed by sequencing of QRDRs)
	methods - analysis of mobile genetic elements	Methods of analysis (e.g. sequencing and comparison to references) of mobile genetic elements (e.g. integrons and/or integron gene cassettes).
	methods - analysis of phenotypic AMR	What methods were applied in the present study for analysis of phenotypic antimicrobial resistance? (e.g. statistical evaluation of AMR between sectors using chi-squared test) *Code newly established at Dhaka (2016).*
	methods - analysis of transfer of (mobile) genetic elements	How did the present study investigate the transferability of mobile genetic elements or ARGs from the isolates included in study (e.g. investigating conjugative transfer of plasmids using conjugation assays/ filter mating)?
	methods - ARGs investigated in study	Which antimicrobial resistance genes were investigated in the study?

	methods - assessment of risk factors associated with AMR bacteria	Study investigated risk factors (epidemiological and statistical investigation) associated with the presence of antimicrobial resistant bacteria (e.g. presence of animal in household).<br>*Code newly established at Lupindu (2015).*
	methods - characterization/analysis of isolates selected in study	methods (e.g. whole genome sequencing(-MLST)/ specific isolation methods/PCR assay for identification of specific serotype of bacteria) used in study
	methods - detection of antibiotic resistance (phenotypic)	Which method(s) were used to assess phenotypic resistance in bacteria in this study? (e.g. disk diffusion method)
	methods - detection of antibiotic resistance genes (genotypic)	Which method(s) were used to assess genotypic resistance in bacteria in this study? (e.g. PCR)
	methods - detection of mobile genetic elements	Methods applied for detection of mobile genetic elements (e.g. PCR for detection of integrons or integron gene cassettes using specific primers).
	methods - general approach	General approach meaning e.g. pangenome approach/metagenomic study.
	methods - identification method of isolates included in study	Identification method of isolates meaning e.g. specific cultivation method or genomic approach.
	methods - inclusion criteria of isolates	Methods used in study for inclusion criteria of isolates (e.g. cut-off value of similarity to bacterial species study is interested in).
	methods - interpretation of antibiotic resistance testing	How did the authors interpret the results of antimicrobial susceptibility testing (e.g. epidemiological cut-off values)? E.g. according to CLSI guidelines for disk diffusion test or definition of MDR<br><br>"The two dominant guiding organizations for standardized susceptibility testing are the Clinical and Laboratory Standards Institute (CLSI) and the European Committee for Antimicrobial Susceptibility Testing (EUCAST)." (Doern, C.D. 2020; Fosfomycin Susceptibility Testing; Clinical Microbiology Newsletter_Vol 42, Issue 4, Pages 27-31)
	methods - MLST	Study using MLST as a method
	methods - mobile genetic elements investigated in study	Which mobile genetic elements (e.g. integrons, gene cassettes) were investigated in the study?
	methods - PGAdb-builder for analysis	Study using PGAdb-builder for analysis
	methods - source tracking/phylogeny	Study using source tracking/ phylogeny (not specifically concerning antibiotic resistance (genes)) as a method. Also

		compromising e.g. PFGE for clonality analysis of isolates.
	methods - special cultivation method of bacteria	This study applied a special method for cultivation of bacteria e.g. using some ABC.
	methods - whole genome MLST schemes	Study specifically using whole genome MLST schemes
results	results - (risk) factors associated with AMR bacteria	Study investigated risk factors (epidemiological and statistical investigation) associated with the presence of antimicrobial resistant bacteria (e.g. presence of animal in household) and presents the results here. Or other factors (e.g. season) associated with AMR (presence/prevalence/distribution/...). *Code newly established at Lupindu (2015).*
	results - AMR bacteria species	Study investigated presence of a certain resistance (geno-/phenotypic, e.g. mcr-1) in samples. Which bacteria species were positive for the resistance investigated?
	results - analysis of phenotypic AMR	Results of analysis of phenotypic antimicrobial resistance? (e.g. statistical evaluation of AMR between sectors using chi-squared test: Significant interference or not?) *Code newly established at Dhaka (2016).*
	results - antibiotic multiresistance	Study investigated multiresistance to antibiotics in bacteria investigated and shows these results. *Code newly established at Busani (2004).*
	results - antibiotic resistance (geno-/phenotypic; all sectors)	Study results concerning geno- and/or phenotypic antibiotic resistance (antibiotic resistance genes/phenotypic resistance) in all sectors/in general (e.g. frequency of MRSA (identification of MRSA using pheno- and genotypic criteria)). *Code newly established at Chao (2013).*
	results - antibiotic resistance (geno-/phenotypic; animal sector)	Study results concerning geno- and/or phenotypic antibiotic resistance (antibiotic resistance genes/phenotypic resistance) in the animal sector (e.g. frequency of MRSA (identification of MRSA using pheno- and genotypic criteria)). *Code newly established at Chao (2013).*
	results - antibiotic resistance (geno-/phenotypic; environmental sector)	Study results concerning geno- and/or phenotypic antibiotic resistance (antibiotic resistance genes/phenotypic resistance) in the environmental sector (e.g. frequency of MRSA (identification of MRSA using pheno- and genotypic criteria)). *Code newly established at Chao (2013).*

	results - antibiotic resistance (geno-/phenotypic; human sector)	Study results concerning geno- and/or phenotypic antibiotic resistance (antibiotic resistance genes/phenotypic resistance) in the human sector (e.g. frequency of MRSA (identification of MRSA using pheno- and genotypic criteria)).<br>*Code newly established at Chao (2013).*
	results - antibiotic resistance (geno-/phenotypic; unknown/various sector)	Study results concerning geno- and/or phenotypic antibiotic resistance (antibiotic resistance genes/phenotypic resistance) in the unknown/various sector (e.g. frequency of MRSA (identification of MRSA using pheno- and genotypic criteria) in food).<br>*Code newly established at Chao (2013).*
	results - antibiotic resistance (genotypic; all sectors)	Results concerning antibiotic resistance investigated in study (genotypic) in all/various sectors.
	results - antibiotic resistance (genotypic; animal sector)	Study results concerning genotypic antibiotic resistance (antibiotic resistance genes) in the animal sector (frequency, detected ARGs, etc.).<br>*Code newly established at Cao (2016).*
	results - antibiotic resistance (genotypic; environmental sector)	Study results concerning genotypic antibiotic resistance (antibiotic resistance genes) in the environmental sector (frequency, detected ARGs, etc.).<br>*Code newly established at Cao (2016).*
	results - antibiotic resistance (genotypic; human sector)	Study results concerning genotypic antibiotic resistance (antibiotic resistance genes) in the human sector (frequency, detected ARGs, etc.).<br>*Code newly established at Cao (2016).*
	results - antibiotic resistance (genotypic; unknown sector)	Study results concerning genotypic antibiotic resistance (antibiotic resistance genes) in the unknown/various sector/s (e.g. foodborne isolates with isolates from raw milk, not mentioned separately) (frequency, detected ARGs, etc.).<br>*Code newly established at Cao (2016).*
	results - antibiotic resistance (phenotypic; all sectors)	Results concerning antibiotic resistance investigated in study (phenotypic) in all/various sectors.
	results - antibiotic resistance (phenotypic; animal sector)	Results concerning (phenotypic) antibiotic resistance investigated in animals in this study.
	results - antibiotic resistance (phenotypic; environmental sector)	Results concerning (phenotypic) antibiotic resistance investigated in the environment in this study.
	results - antibiotic resistance (phenotypic; human sector)	Results concerning (phenotypic) antibiotic resistance investigated in humans in this study.
	results - antibiotic resistance (phenotypic; unknown sectors)	Results concerning antibiotic resistance investigated in study (phenotypic) in unknown/various sectors (e.g. "food-borne isolates" including food of animal and unknown origin).

	results - antibiotic resistance genes	Results of antibiotic resistance genes detected in study
	results - comparison of antibiotic resistance (genotypic) between sectors	Study results are comparing ARGs (e.g. in terms of their prevalence and distribution) between different sectors (e.g. human, animal and environment or only two of those).<br>*Code newly established at Cao (2016).*
	results - comparison of antibiotic resistance (phenotypic) between sectors	Study compares antibiotic resistance between two or three sectors (environment, human, animal). This does not implicate that the study investigated (routes/directions) spread of antimicrobial resistance and phlyogeny/source tracking.
	results - comparison of methods for detection of AMR (phenotypic/genotypic)	Study compares different methods for detection of AMR (genotypic and/or phenotypic resistance) e.g. in terms of sensitivity.
	results - documentation of antibiotic resistance (geno- or phenotypic) according to origin	Does the study distinguish between the origin of the samples concerning the grade of antibiotic resistance? Or does it finally just have a look at the overarching antibiotic resistance (putting all samples/isolates into one pot)?
	results - further analysis of ARGs	Study mentions results of further analysis of ARGs (e.g. sequencing of RT-PCR products for investigation of matching nucleotide sequences to known sequences for certain ARGs).
	results - indicating host specificity concerning AMR	Study results indicate host specificity of antimicrobial resistance (either pheno- or genotypic). For example an ARG that was exclusively or more often found in a certain sector (e.g. environment). This could be an indication that there is no/only limited spread of AMR between sectors.<br>*Code newly established at Cao (2016).*
	results - mechanisms of resistance	Which mechanisms of resistance were detected in the study and made responsible for the resistance observed? (e.g. mutations in some resistance-determining regions of ARGs) What are the causes of the antibiotic resistance observed?
	results - methods for isolation of ABR bacteria/ARGs	Study established/discussed a (new) method for isolation of antibiotic resistant organisms (pheno- and/or genotypic). E.g. study showing results that special cultivation methods are better for isolation/detection of mcr-1 positive bacteria than PCR detection methods.
	results - mobile genetic elements	Results concerning mobile genetic elements (prevalence, etc.).
	results – mobile genetic elements differentiating between sectors	Results concerning mobile genetic elements (prevalence, etc.) differentiating between the sectors (e.g. human vs. animal).

	results - source tracking with evidence of spread	Study gives some evidence of or at least suggests spread of antimicrobial resistance (genes) between sectors (e.g. human and environment).
	results - source tracking with no evidence of spread	Study shows evidence or suggests no spread between the sectors (supporting our hypothesis that the 3 sectors are quite separated in terms of antimicrobial resistance (genes) and spread does not occurr frequently)
	results - source tracking/phylogeny	results meaning data generated in study, source tracking meaning investigating phylogenetic relationships not necessarily linked to or concerning antibiotic resistance (genes)
	results - source tracking/phylogeny antibiotic resistance	Source tracking/phylogeny analysis performed in study, specifically concerning antibiotic resistance (genes) (including mobile genetic elements)
	results - transfer of (mobile) genetic elements	Results concerning transferability of mobile genetic elements from the isolates included (e.g. results of conjugative transfer of plasmids using conjugation assays).
discussion	discussion - (outlook for) study design	Study describes outlook for better study design (e.g. sample size, further investigations to be done to answer some questions remaining unclear). Or study already applied a certain method that seems to be a good idea for further studies (e.g. selection of ABC being investigated in susceptibility testing based on epidemiologic data instead of chosing them randomly).<br>*Code newly established at Deredjian (2011).*
	discussion - (probable) factors associated with AMR	What are (probable) factors associated with the arise or/and spread of AMR? E.g. exposition to ABC as a factor.<br>*Code newly established at Field, W. 2015.*
	discussion - development of ABR-situation over years	Study discusses how antimicrobial resistance situation has developed over the last years (e.g. increasing ABR over last 5 years or outlook towards future situation).
	discussion - differences in AMR between geographical regions	Study discusses/shows that there are different prevalence of AMR bacteria depending on geographical regions. (e.g. more ABR in northern areas of a country).
	discussion - probable causes for bias concerning ARGs/AMR	Study discusses probable/plausible causes/sources of bias concerning the detection of ARGs (e.g. storage instability when refrigerated).
	discussion - probable causes of AMR and its spread	Study reflects about probable/possible causes of AMR and its spread. These reflections could provide important clues

		for strategies to try to reduce/control AMR and its spread.
	discussion - recommendations for control of AMR	Study mentions recommendations to control AMR and its spread.
open questions	open questions/information – mobile genetic elements	Study providing information (e.g. results from previous studies) or mentioning open questions concerning mobile genetic elements.
	open question/information - supporting OneHealth approach	Study mentioning and supporting OneHealth approach in terms of solving problem of antimicrobial resistance.
	open questions - drivers of AMR	Study reveals open questions concerning major drivers in AMR (and its spread).
	open questions - phylogenic investigations	Study mentions that further studies should be done investigating phylogeny of bacteria for a better epidemiological understanding (e.g. of distribution in the different sectors).<br>*Code newly established at Hayford (2015).*
	open questions - reasons for phenotypic resistance displayed	Study leaves question open for reasons why isolates investigated show certain phenotypic resistance pattern: What is the genotypic background leading to this? (e.g. efflux pump mechanisms)
	open questions - spread of antimicrobial resistant organisms	Open questions mentioned in study concerning spread of antimicrobial resistant bacteria.
study power (ranking)	study power - bias	Indicating possible source of bias in study. Having negative effect on power of study (e.g. selection bias) or only including e.g. isolates resistant to a certain antibiotic on purpose (e.g. quinolone-resistant isolates).
	study power - potential sources of bias	Study discusses potential sources of bias (e.g. how presence of ARGs should be interpreted, for example that presence alone is not enough to tell if the ARGs are of clinical relevance) in the present study or other studies.<br>*Code newly established at Fitzpatrick, D. and F. Walsh (2016).*
	study power - strength of study (concerning antibiotic resistance)	Highlighting something that gives the present study a higher ranking/more strengh (e.g. high number of samples included, time- and place-matched samples included).
	study power - weaknesses of study (concerning antibiotic resistance)	Weakness of the study discussed in the study itself (e.g. sample size, methods for improving detection of antimicrobial resistance (genes)). Important for our ranking of study power.
obstacles	obstacles – methods for investigating AMR	What are obstacles/limitations concerning the methods making it difficult to detect

What are obstacles in science concerning investigation of AMR and its spread?		AMR (geno- or phenotypic)? (e.g. standard PCR methods often not able to amplify ARGs in gene cassette regions of class 1 integrons).
sources	sources – source that may be read concerning transmission of AMR	
	sources – source to be read concerning transmission of AMR?	
quotes	-	-
important<br>*Important information provided by study concerning our question (knowledge on spread of AMR between sectors/responsibilities/scientific evidence concerning spread of AMR).*<br><br>*Code estalished at Chenk, K (2017). Maybe read those papers before again and screen them for these criteria (to make sure that no information gets lost - some important comments by meiehe were mentioned in those studies).*	important - concerning our hypothesis (spread of AMR between sectors)	Important information concerning our hypothesis: Current knowledge of AMR in the different sector and its spread between the sector.<br>*Code newly established at Chen, K. (2017).*
	important - concerning our hypothesis in a wider sense	Study shows results concerning spread of (AMR) bacteria between sectors, making a link to spread of AMR in a wider context.<br>E.g. the study investigated genetic relatedness between isolates of different sectors using PFGE and showed clustering/high genetic relatedness between different sectors. Lateron in study they demonstrated AMR among those isolates (but did not prove spread of AMR from one sector to another between those isolates).
	important - direct comparison of AMR between sectors	Study provides scientifically founded information about resistance to antibiotics compared between sectors (e.g. statistical analysis for resistance pattern among three sectors).
	important - few scientific evidence indicating transmission of AMR (pheno- or genotypic) between sectors	Study has few scientific evidence (e.g. über verschiedene Ecken) indicating (no) transmission/spread of AMR between sectors.
	important - no scientific evidence but mentioning (possible) spread	Study mentions the topic of spread/transmission of AMR bacteria but has no scientific data supporting this hypothesis.<br>*Code newly established at Chen, K (2017).*
	important - quite good scientific evidence indicating spread of AMR between sectors	Study provides quite good scientific evidence indicating spread (or no spread) of antimicrobial resistance between sectors (e.g. phylogenetic analysis using PFGE of isolates and association with antibiogram profiles).
exclusion	Exclusion criteria HM	Reason why this study should be excluded from our work (e.g. one sector is lacking, etc.).

Exclusion after reading paper (e.g. not all sectors represented).		
	Thoughts HM	Code used to link memos to quotations

Table S6. *Established Code Groups and Codes in Atlas.ti8*

3. Investigated fields: Which animals, environments and humans are known so far? (Animal species, type of environment, geographical location (country))

General remark: In the figures and detailed analysis of the studies included there may be multiple references (Mehrfachnennung) since some studies investigated more than one sector.

Ecosystem interactions
Since we had too many inclusions when selecting publications investigating two of the three compartments (animal and human or human and environment or animal and environment), we decided to further limit our selecting criteria on publications looking at all three compartments/compartments (animal, environment and human). This is also in concordance with our aim to assess the current knowledge on spread of antimicrobial resistance involving all compartments.

Animal species
51 different defined animal species and products (excluded animal samples/isolates of unknown species and metagenomic datasets) were investigated in the studies included in our work. The most prevalent sector investigated were animal products (41 studies (n); e.g. meat, chicken eggs, dairy products), followed by livestock birds (n=34; chicken, duck, turkey, goose, ostriches, Hoatzin), pigs (n=32) and cattle (n=26) (**Figure S1**).

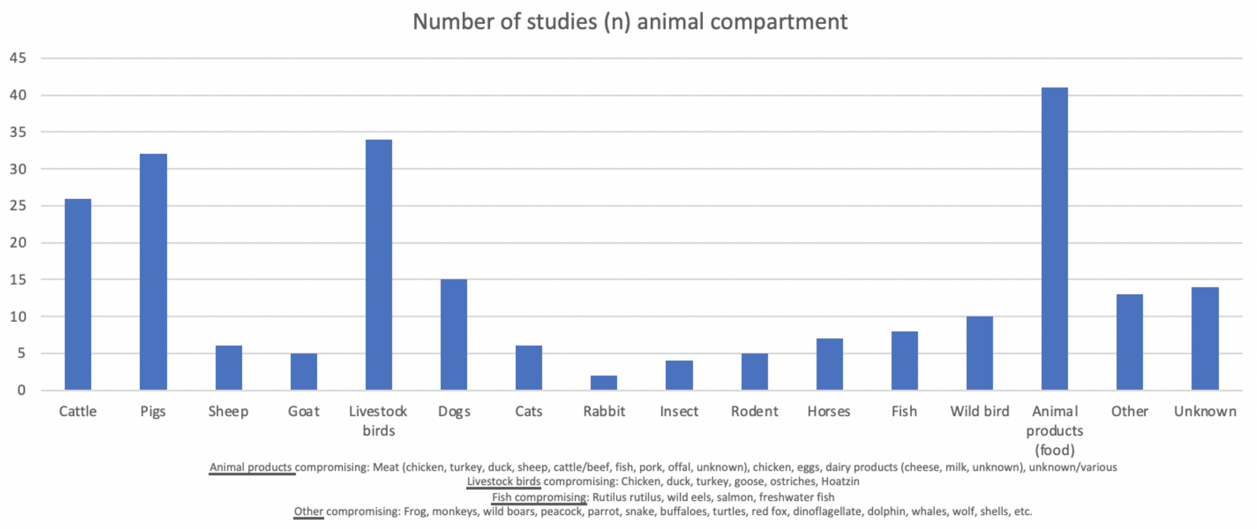

Figure S1. *Types of animal sectors investigated in studies included in our systematic review (number of studies (n)).*

The largest represented animal sector (food of animal origin) consists of 14 different animal species (excluded food of unknown animal source and metagenomic datasets) that were investigated in studies included in our work (**Figure S2**).

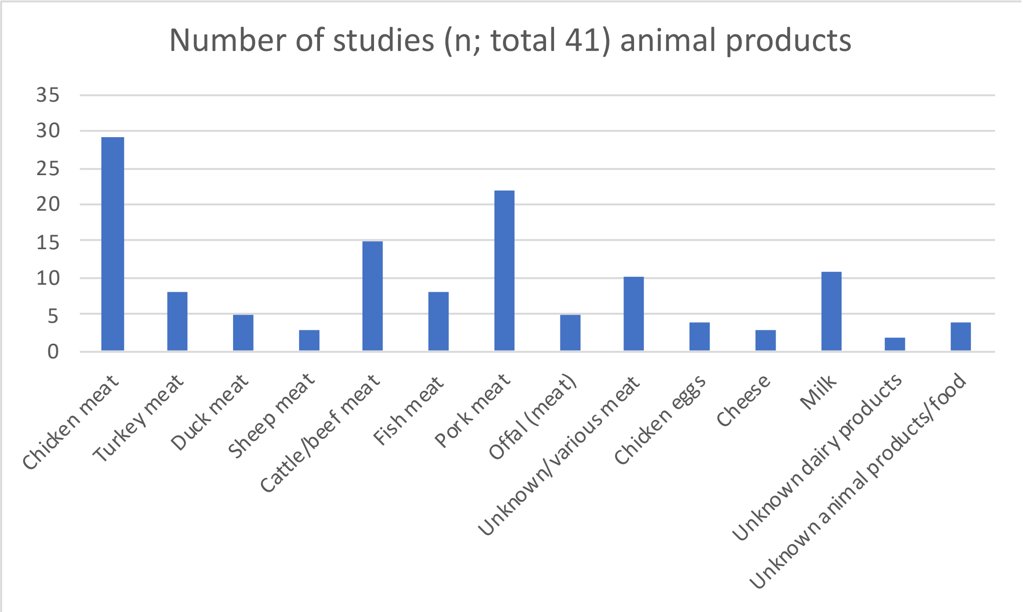

Figure S2.*Types of animal products/food of animal origin represented in the 41 studies included in our systematic review investigating this compartment (number of studies (n)).*

Type of environment

In total 13 defined environmental niches (excl. unknown environments and metagenomic datasets) were investigated (**Figure S3**), the water sector being the most represented in all included studies. Wastewater and river water were the most prevalent within the water sector.

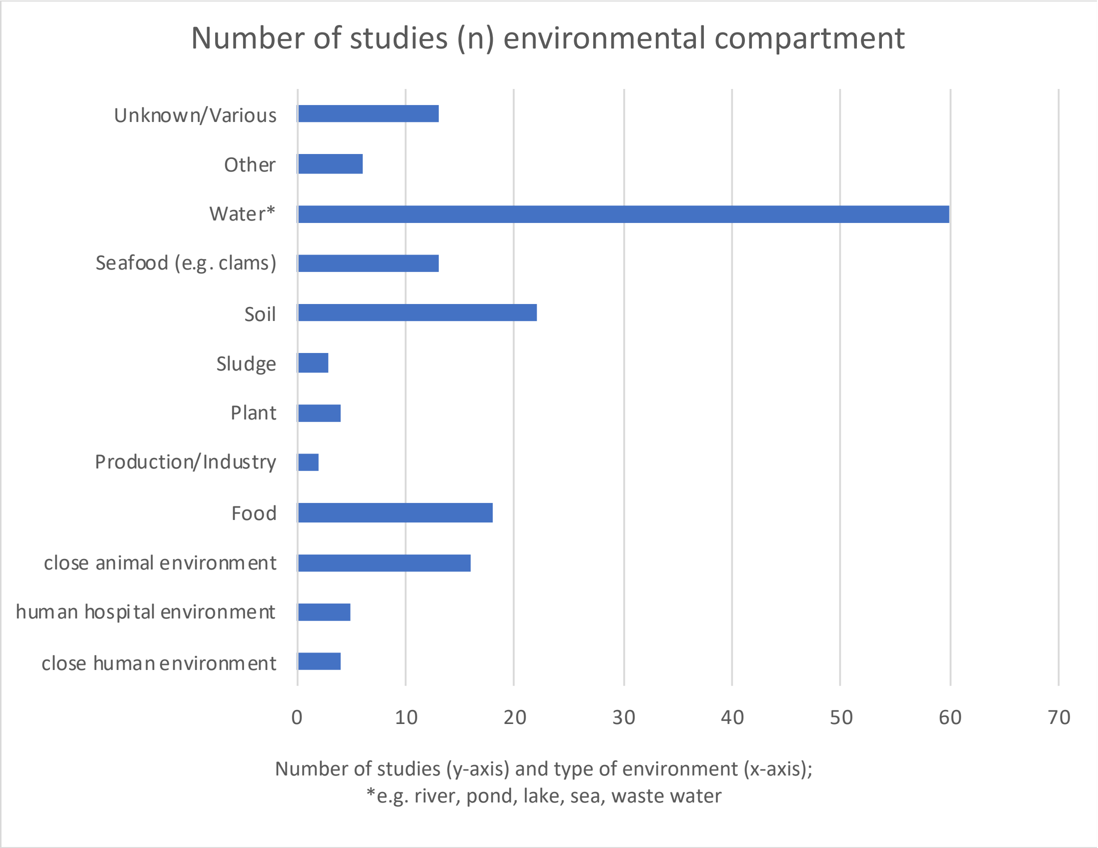

Figure S3. *Environmental niches represented in our systematic review (number of studies (n)).*

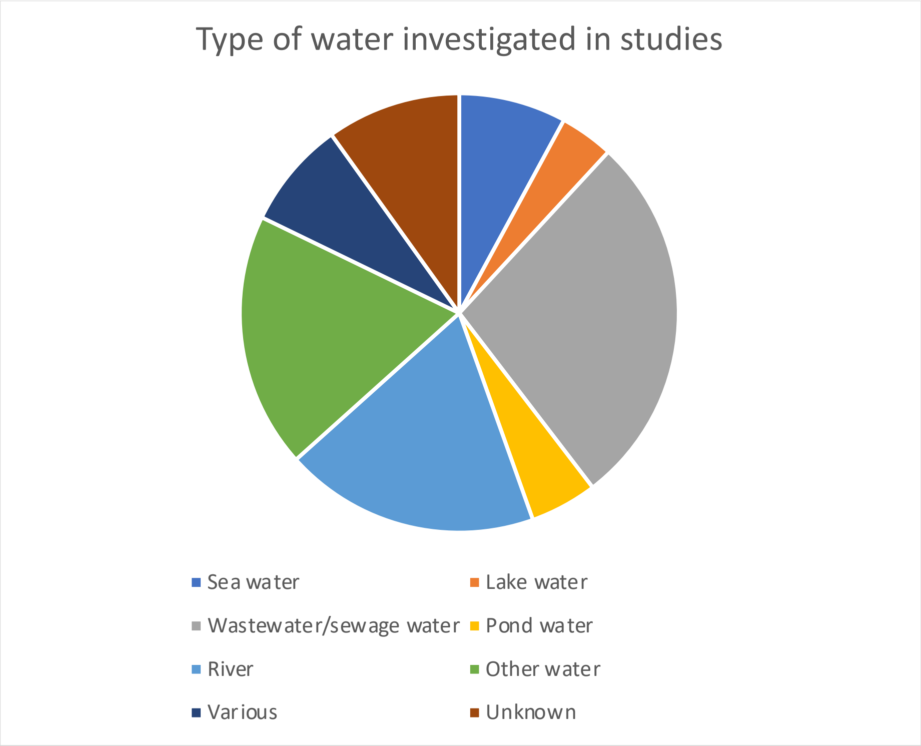

Figure S4. *Types of water sources represented in the 60 studies investigating water environment included in our systematic review.*

Geographical representation

A total of 92 different countries were represented in the studies (excluded metagenomic datasets and unknown geographical origin of samples), China and the US being the main sources of isolates investigated in the studies (**Figures S5** and **S6**).

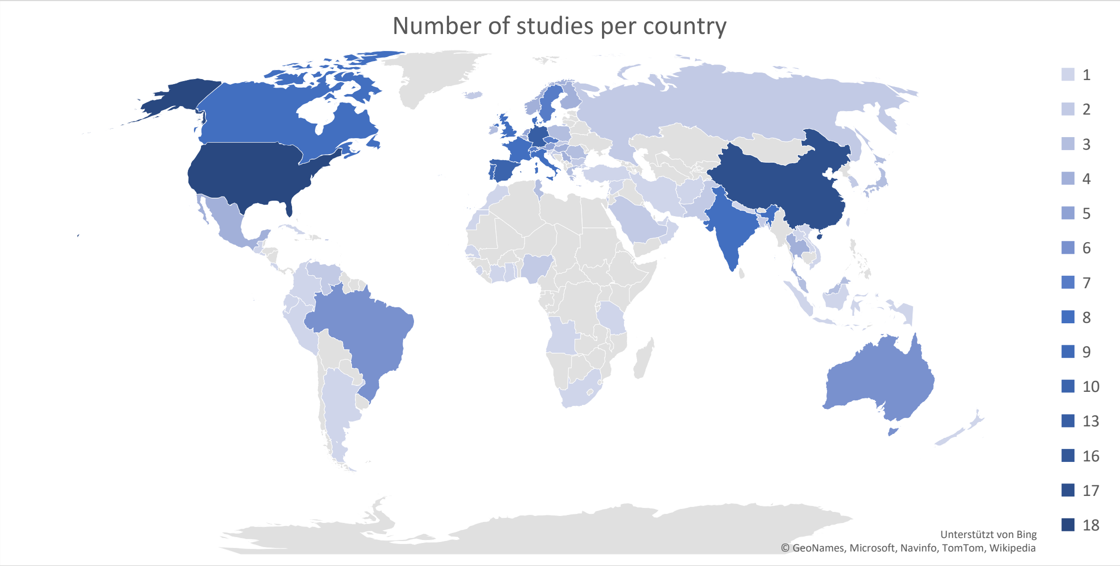

Figure S5. *Countries represented in studies included in our systematic review.*

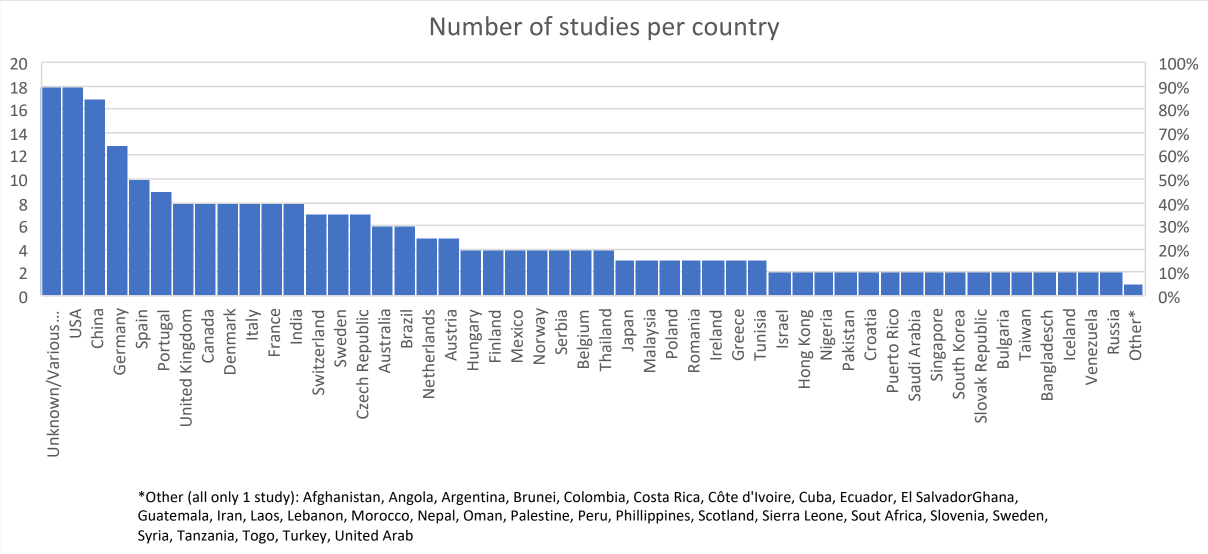

Figure S6. *Countries only represented once in included studies are summarized in group "other".*

Human population

A large majority of studies investigated clinical samples of the human population.

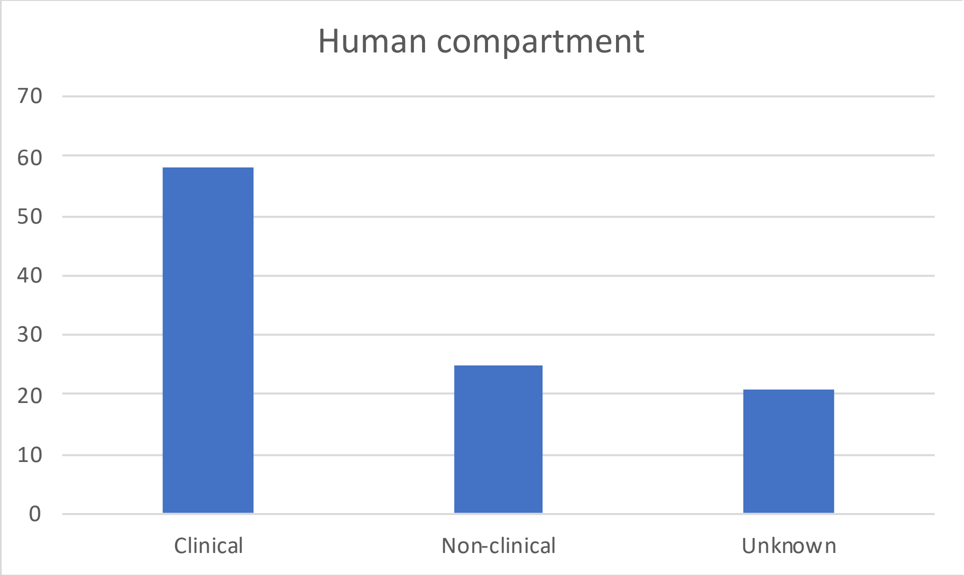

Figure S7. *Human compartment divided into clinical, non-clinical and unknown.*

4. Investigated bacteria and resistance genes: Which bacteria (taxonomy) and resistance genes were investigated? Which classes of antimicrobial agents were investigated in the resistance testing?

General remark: In the detailed analysis of the studies included there may be multiple references since some studies investigated more than one bacteria.

The distinction and classification of bacteria into gram-negative, -positive and -indeterminate according to their different cell wall structure is being used for practical purposes in this chapter.

Gram-negative bacteria
In the studies (n) included in our work the following gram-negative bacteria were investigated:
Phylum Proteobacteria (n=60) with the following classes and species:
- Alpha-Proteobacteria (n=1): *species unknown* (n=1)
- Beta-Proteobacteria (n=1): *species unknown* (n=1)
- Gamma-Proteobacteria (n=55): *Acinetobacter baumannii* (n=1), *Aeromonas spp.* (n=3), *Escherichia coli* (n=14), *Escherichia coli* and *Salmonella enterica* (n=1), *Enterobacteriaeceae* (n=3), commensal coliforms [*Escherichia coli and non-E. coli coliforms (Citrobacter freundii, Enterobacter cloacae, Klebsiella pneumoniae, Salmonella enteritidis, Shigella flexneri;* n=1], ? (n=1), *Plesiomonas shigelloides* (n=1), *Pseudomonas aeruginosa* (n=2), *Salmonella enterica* (n=4), *Salmonella* spp. (n=5), *Salmonella enterica* serotype Infantis (n=1), *Salmonella enterica subsp. enterica serovar 4,[5],12:i:- (monophasic variant of Salmonella typhimurium)* (n=2), *Salmonella enterica* (serotype 1,4,[5],12:i:-R-Type ASSuT; n=1), *Salmonella* enterica serovar Saintpaul (n=1), *Salmonella enterica* serovar *Enteritidis* (n=1), *Salmonella enterica* Serovar Bovismorbificans (n=1), *Salmonella enterica serovar Newport* (n=1), *Salmonella enterica* serovar Stanley (n=1), *Salmonella enterica* serovar Typhimurium (n=1), AMR nontyphoidal *Salmonella* (n=1), *Salmonella enterica* serovar paratyphi B (n=1), *Serratia marcenscens* (n=1), *Vibrio cholerae* (n=1), Enteropathogenic *Yersinia spp.* (n=1)
- Delta-Proteobacteria (n=1): *species unknown* (n=1)
- Epsilon-Proteobacteria (n=2): *Campylobacter jejuni* (n=1), *Campylobacter spp.* (n=1),

Gram-positive bacteria
In the studies (n) included in our work the following gram-positive bacteria were investigated:
Phylum Firmicutes (n=17) with the following classes:
- Bacilli (n=5): *Bacillus cereus* (n=1), *Listeria spp.* (n=1), *Listeria spp.* (*L. monocytogenes, L. innocua, L. welshimeri*; n=1), *Listeria monocytogenes* (n=2)
- Clostridia (n=3): *Clostridium perfringens* (n=2), *Clostridium difficile* (RT078 strains; n=1)
- Diplococci (n=9): *Enterococci spp.* (n=6), *Enterococcus faecium* (n=3)

Phylum Actinobacteria (n=1)

Gram-indeterminate bacteria
In the studies (n) included in our work the following gram-indeterminate bacteria were investigated:
Phylum Actinobacteria (n=1):
- *Mycobacterium spp.* (n=1)

Summary of investigated phyla of bacteria
In total 15 different bacteria genera (**Figure S8**) of 3 different phyla (Actinobacteria, Firmicutes, Proteobacteria; **Figure S9**) were studied in papers included into the systematic review (excl. metagenomic datasets and not specified/unknown).
The main studied bacteria phylum included in this work was the phylum Proteobacteria (60 studies). The phylum Firmicutes was investigated in 17 studies, the phylum Actinobacteria in 2 studies. Therefore 60 studies investigated gram-negative bacteria, 18 studies gram-positive bacteria and 1 study gram-indeterminate bacteria (Mycobacteria).
There were some studies e.g. using a metagenomic approach that looked at multiple bacterial phyla which are not part of the following table (see **Table S7**).

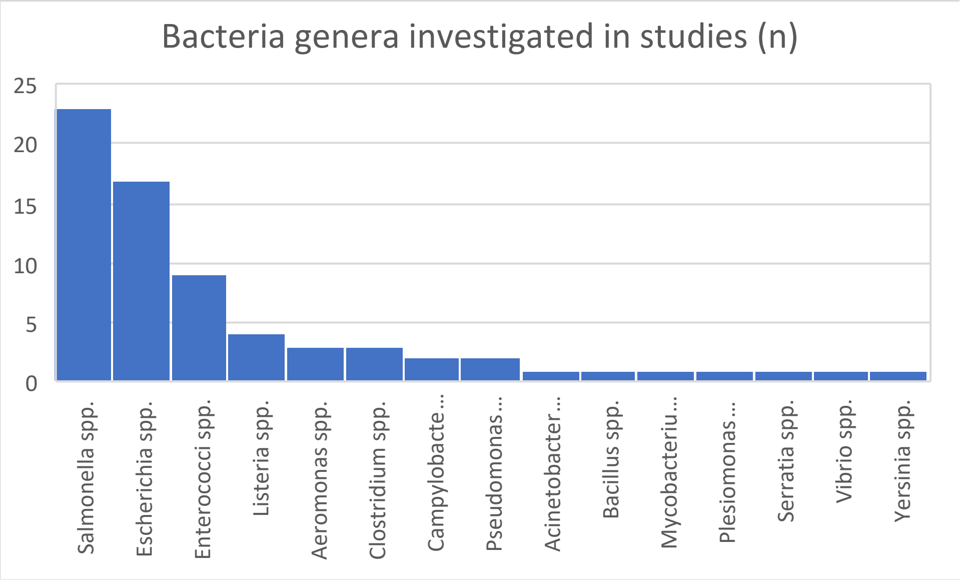

Figure S8. *Bacteria genera investigated in studies (n).*

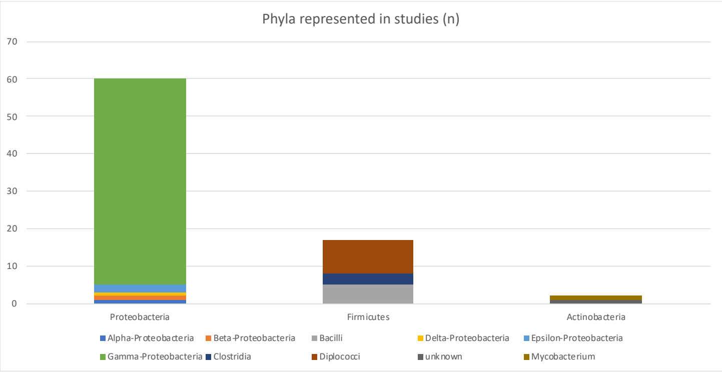

Figure S9. *Bacteria phyla represented in studies (n).*

Overview of bacteria represented in included studies
The following bacteria are represented in the included studies (n):

**Genus (spp.)**	**Specific bacteria species/serovar**	**Gram/Phylum**
Acinetobacter spp. (n=1)	*Acinetobacter baumannii* (n=1)	Proteobacteria (class: Gamma-Proteobacteria), gram-negative
Aeromonas spp. (n=3)	Aeromonas spp. (n=3)	Proteobacteria (class: Gamma-Proteobacteria), gram-negative
Bacillus spp. (n=1)	*Bacillus cereus* (n=1)	Firmicutes (class: Bacilli), gram-positive
Campylobacter spp. (n=2)	- *Campylobacter jejuni* (n=1)<br>- *Campylobacter spp.* (n=1)	Proteobacteria (class: Epsilonproteobacteria), gram-negative
Clostridium spp. (n=3)	- *Clostridium perfringens* (n=2)<br>- *Clostridium difficile* (RT078 strains) (n=1)	Firmicutes (class: Clostridia), gram-positive
Enterococci spp. (n=9)	- *Enterococci spp.* (n=6)<br>- *Enterococcus faecium* (n=3)	Firmicutes (class: diplococci), gram-positive
Escherichia spp. (n=16)	*Escherichia coli* (n=13)	Proteobacteria (class: Gammaproteobacteria), gram-negative
Listeria spp. (n=4)	- *Listeria spp.*<br>- *Listeria monocytogenes, Listeria innocua, L. welshimeri*<br>- *Listeria monocytogenes* (n=2)	Firmicutes (class: Bacilli), gram-positive
Metagenomic study (n=10)		
Multiple bacteria analyzed in one study (n=9)	- *Escherichia coli* and *Salmonella enterica* (n=2)<br>- *Enterobacteriaceae* (n=3)<br>- *mcr-1*-bearing gram-negative bacteria<br>- Gram-negative bacteria with focus on *ESBL-producing Escherichia coli*<br>- Commensal coliforms [*Escherichia coli and non-E. coli coliforms (Citrobacter freundii, Enterobacter cloacae, Klebsiella pneumoniae, Salmonella enteritidis, Shigella flexneri*]<br>- Bacterial genera carrying aac(6′)-Ib and ist variants (34 genera; 5 different classes: 4 Actinobacteria, 2 Alphaproteobacteria, 32 Betaproteobacteria, 1 Deltaproteobacteria, 3925 Gammaproteobacteria)	- Proteobacteria (class: Gammaproteobacteria), gram-negative<br>- Proteobacteria (class: Gammaproteobacteria), gram-negative<br>- ?, gram-negative<br>- i.e. Proteobacteria (class: Gammaproteobacteria), gram-negative<br>- Proteobacteria (class: Gammaproteobacteria), gram-negative<br>- Actinobacteria, gram-positive; Proteobacteria (classes: Alpha-, Beta-, Delta-, Gamma-proteobacteria), gram-negative
Mycobacterium spp. (n=1)	*Mycobacterium spp.* (rapidly growing) (n=1)	Actinobacteria, acid fast (not well stainable by Gram stain procedure)
Plesiomonas Shigelloides (n=1)	*Plesiomonas shigelloides* (n=1)	Proteobacteria (class: Gammaproteobacteria), gram-negative

Pseudomonas spp. (n=2)	*Pseudomonas aeruginosa* (n=2)	Proteobacteria (class: Gammaproteobacteria), gram-negative
Salmonella spp. (n=22)	- *Salmonella enterica* (n=4)<br>- *Salmonella* spp. (n=5)<br>- *Salmonella enterica* serotype Infantis<br>- *Salmonella enterica subsp. enterica serovar 4,[5],12:i:- (monophasic variant of Salmonella typhimurium)* (n=2)<br>- *Salmonella enterica* (serotype 1,4,[5],12:i:-R-Type ASSuT)<br>- *Salmonella* enterica serovar Saintpaul<br>- *Salmonella enterica* serovar *Enteritidis*<br>- *Salmonella enterica* Serovar Bovismorbificans<br>- *Salmonella enterica serovar Newport*<br>- *Salmonella enterica* serovar Stanley<br>- *Salmonella enterica* serovar Typhimurium<br>- AMR nontyphoidal *Salmonella*<br>- *Salmonella enterica* serovar paratyphi B	Proteobacteria (class: Gammaproteobacteria), gram-negative
Serratia spp. (n=1)	*Serratia marcenscens* (n=1)	Proteobacteria (class: Gammaproteobacteria), gram-negative
Vibrio spp. (n=1)	*Vibrio cholerae* (n=1)	Proteobacteria (class: Gammaproteobacteria), gram-negative
Yersinia spp. (n=1)	Enteropathogenic *Yersinia* spp.	Proteobacteria (class: Gammaproteobacteria), gram-negative

Table S7. *Overview of bacteria represented in studies included.*

Classification of antibiotic resistances

Phenotypic resistance: Antimicrobials investigated in included studies
The total number of different groups of antibiotics investigated in the included studies was 24 (excluding others).

Antibiotic group	Antibiotic	Number of studies (n)
ansamycin	rifampicin (rifamycin)	8
	rifampin (semisynthetic derivative of rifamycin)	4
aminoglycoside	amikacin	26
	apramycin	1
	gentamicin	51
	isepamicin	2
	kanamycin	25
	neomycin	5
	netilmicin (sulfate)	5
	sisomicin	1
	spectinomycin	3
	streptomycin	33
	tobramycin (= tobramycin)	8
	aminoglycoside (unknown)	1
β-lactam	amdinocillin (= mecillinam; extended spectrum penicillin)	1
	ampicillin (aminopenicillin)	49
	ampicillin (aminopenicillin; in combination with sulbactam)	2
	ampicillin (aminopenicillin; in combination with clavulanic acid)	1
	ampicillin A	1
	amoxicillin (aminopenicillin)	3
	amoxicillin (aminopenicillin; in combination with clavulanic acid (n=27) or clavulanate (n=1))	28
	amoxicillin (aminopenicillin; in combination with sulbactam)	1
	aztreonam (monobactam)	11
	carbenicillin (carboxypenicillin)	1
	piperacillin (acylamino-penicillin; in combination with tazobactam)	12
	piperacillin (acylamino-penicillin, alone)	6
	penicillin	5
	benzylpenicillin (penicillin G)	3
	mezlocillin (ureido-/acylaminopenicillin)	2

	cefalexin (cephalosporin)		1
	cefamandole (2nd generation cephalosporin)		1
	cefazolin (1st generation cephalosporin)		6
	cefepime (4th generation cephalosporin)		17
	cefixime (3rd generation cephalosporin)		2
	cefoperazone (3rd generation cephalosporin; in combination with sulbactam)		2
	cefoperazone (3rd generation cephalosporin; alone)		3
	cefoxitin (2nd generation cephalosporin)		19
	ceftazidime (3rd generation cephalosporin)		25
	cefuroxime (2nd generation cephalosporin)		6
	cephalothin (1st generation cephalosporin)		10
	ceftriaxone (3rd generation cephalosporin)		19
	ceftiofur (3rd generation cephalosporin)		11
	cefotaxime (3rd generation cephalosporin; alone)		32
	cefotaxime (3rd generation cephalosporin; in combination with clavulanate)		1
	cefpodoxime (3rd generation cephalosporine)		6
	cephalosporine (unknown)		1
	carbapenem	ertapenem	5
		imipenem	19
		meropenem	14
		carbapenem	2
	oxacillin (isoxazolylpenicillin)		3
	ticarcillin (carboxypenicillin; alone)		6
	ticarcillin (carboxypenicillin; in combination with clavulanic acid)		2
β-lactamase inhibitors	clavulanic acid (in com-bination with amoxicillin)		27
	clavulanic acid (in combination with ampicillin)		1

	clavulanate (in combination with cefotaxime (n=1) or amoxicillin (n=1))	2
	tazobactam (in combination with piperacillin)	12
	sulbactam (in combination with cefoperazone)	2
	sulbactam (in combination with ampicillin)	2
	sulbactam (in combination with amoxicillin)	1
cyclic lipopeptide	daptomycin	2
pyrimidine	methoxybenzyl aminopyrimidine	1
	trimethoprim (diaminopyrimidine; in combination with sulfamethoxazole)	45
	trimethoprim (diaminopyrimidine; alone)	7
epoxide	fosfomycin	8
fenicol	chloramphenicol	40
fusidane	fusidic acid	1
glycopeptide	teicoplanin	7
	vancomycin	14
glycylcycline	tigecycline	10
fluorquinolone	ciprofloxacin	57
	danofloxacin	1
	enrofloxacin	5
	levofloxacin (3rd generation fluorquinolone)	11
	moxifloxacin (4th generation fluorquinolone)	3
	ofloxacin	7
quinolone	nalidixic acid	35
	oxolinic acid	3
	pefloxacin	1
	flumequine	3
	gatifloxacin	2
	norfloxacin	6

lincosamide	clindamycin	6
	lincomycin	1
macrolide	azithromycin	1
	clarithromycin	1
	erythromycin	18
	tylosin	1
nitrofuran	nitrofurantoin	10
	furazolidone	1
nitrobenzol derivates	florfenicol	4
nitroimidazole	metronidazol	1
oxazolidinone	linezolid	6
	tedizolid	1
polypeptide	colistin (= polymyxin E; polymyxin; and colistin sulfate)	9
	polymyxin B	1
sulfonamide	sulfisoxazole	6
	sulfadimethoxine	1
	sulfamethoxazole (in combination with trimethoprim)	45
	sulfafurazole	1
	sulfamethoxazole (alone)	4
	(compound) sulfonamides	8
streptogramin	dalfopristin (in combination with quinupristin)	4
	quinupristin (in combination with dalfopristin)	4
tetracycline	chlortetracycline	2
	doxycyclin	3
	tetracycline	56
	minocycline	3
	oxytetracycline	4
quinoxaline-di-N-oxide	carbadox (carbamate + chinoxalin derivate)	1
	mequinox	1
	olaquindox	1

Other	phenylalanine-arginine-β-naphthylamide (efflux pump inhibitor)	1
	muciprocin (=mupirocin)	1

Table S8. *Antimicrobials investigated in included studies (n)*

Genotypic resistance: Classification of mobile genetic elements and antimicrobial resistance genes and their encoding resistance represented in included studies

Antimicrobial resistance genes	
**Antimicrobial resistance encoding gene**	**Antimicrobial resistance (mechanism) encoded**
*amp*C	Beta lactamase/Beta lactams
blaCARB	
blaCTX	
blaCTX-M	
blaCTX-M-1	
blaCTX-M-2	
blaCTX-M-8	
blaCTX-M-9	
blaCTX-M-15	
blaCMY (plasmid-mediated AmpC (pAmpC))	
blaCMY-2 (ceftiofur, ceftriaxone resistance)	
blaNDM (carbapenem resistance)	
blaNDM-1 (carbapenem resistance)	
blaTEM (resistance to ampicillin/amoxicillin)	
blaTEM-1	
blaOXA	
blaOXA-1	
blaOXA-1-like	
blaOXA-23/-24	
blaOXA-58	
blaOXY	
bla_1 SVH prec	
blaPSE(-1) (resistance to ampicillin)	
blaSHV	
blaSHV-2	
blaSHV-5	
blaACC (family-specific plasmid-mediated AmpC beta-lactamase gene)	
cfxA	
CIT (family-specific plasmid-mediated AmpC beta-lactamase gene)	
DHA (family-specific plasmid-mediated AmpC beta-lactamase gene)	
EBC (family-specific plasmid-mediated AmpC beta-lactamase gene)	
FOX (family-specific plasmid-mediated AmpC beta-lactamase gene)	
MOX (family-specific plasmid-mediated AmpC beta-lactamase gene)	
kpc precursor	

smeA	
mrdA	
blaA	
blaB	
*bla*TEM-1 (class A beta-lactamase)	
*bla*VEB	
*bla*GES	
*bla*PER	
*bla*VIM (carbapenem resistance)	
*bla*IMP (carbapenem resistance)	
*bla*KPC	
mecA	beta lactams (PBP-2a)
pbp5	ampicillin
mdfA	multidrug resistance
mdtA (aminocoumarin resistance)	
mdtA-1	
mdtB (aminocoumarin resistance)	
mdtB-1	
mdtB-2	
mdtC (aminocoumarin resistance)	
mdtC-2	
mdtE	
mdtF	
mdtG	
mdtK	
mdtM	
mdtN	
mdtO	
mdtP	
robA	
AcrA	
AcrB	
AcrE	
AcrF	
baeR	
baeS	
Bcr	
cpxA	
cpxR	
CRP	
EmrD	
evgS	

alaS	aminocoumarin
cysB	
acrD	
aac(3)IIa	aminoglycosides
aacA-aphD	
aacA4 (aminoglycoside acetyltransferase)	
aadA1 *(=aadA=ant(3'');streptomycin resistance; gene cassette)*	
aadA2 (streptomycin resistance; gene cassette)	
aadD	
aadE	
apmA	
aphA (aminoglycoside phosphotransferase)	
aphA3 (aminoglycoside phosphotransferase)	
aph(2")-Ia-acc(6')-Ie	
aph(2")-Ib (=*aph(2")-IIa*)	
aph(2")-Ic	
aph(2")-Id	
aph(3)IIa	
aph(3')III	
aph(3')IIIa	
aph(3")-IIIa	
aac6	
aac(3) -IV	
aac(3)-IIa	
aac(6')-Ib (aminoglycoside N-acetyltransferase conferring resistance to tobramycin, kanamycin and amikacin))	
AAC(6')-Ie-APH(2'')-Ia (aminoglycoside acetyltransferase encoded by plasmids and transposons)	
aac(6')-Ie/aph(2")	
ant(2'')-Ia (= aadB; gentamicin, kanamycin)	
ant(3")-Ia *(aadA)*	
ant(4', 4'')	
ant(4')-Ia	
ant(6)Ia (*aad(6)*)	
ANT(6)-Ib	
ANT(9)-Ia (aad(9))	
neo (aminoglycoside phosphotransferase)	
npmA	
rmtB (16S rRNA methyltransferase)	

strA (streptomycin resistance; = *aph(3'')-Ib*)	
strB (streptomycin resistance; = *aph(6)-Id*)	
strA-strB (streptomycin resistance)	
str (?)	
kpdE	
tolC	
cat1 (chloramphenicol acetyltransferase)	chloramphenicol
cat2 (chloramphenicol acetyltransferase)	
*cat(*pC221*)* (A-7) (chloramphenicol acetyltransferase)	
*cat(*pC223*)* (A-8) (chloramphenicol acetyltransferase)	
*cat(*pC194*)* (A-9) (chloramphenicol acetyltransferase)	
cat86 (A-6) (chloramphenicol acetyltransferase)	
catS (A-12) (chloramphenicol acetyltransferase)	
catDP (A-11) (chloramphenicol acetyltransferase)	
catB (A-15) (chloramphenicol acetyltransferase)	
catQ (A-16) (chloramphenicol acetyltransferase)	
mdtL	
flo	
floR	
cml	
cmlA	
cmlA1	
cmlB	
catA	
catA1	
cat3	
cfr	phenicol, oxazolidinone, lincosamid, macrolide, streptogramin (Cfr rRNA methyltransferase)
cfr1	
cfr(B)	
mupA	mupirocin
arnA	
arnC	
arnT_2 (polymyxin B)	
arnE (polymyxin B)	polymyxin

mcr-1	
PmrB	
PmrC	
PmrE	
bacA	bacitracin
bacA_1	
uppP	
sat (e.g. sat-4)	streptogramin
hslJ	novobiocin
rpsL	streptomycin
leuO	sulfonamides
sul1	
sul2 (sulfamethoxazole)	
sul3	
sul(A)	
aacC2	Gentamicin (aminoglycoside), kanamycin
aacC4	
aphA-1, aphA	Kanamycin
aphA2	
Kn	
dfrA	trimethoprim
dfrA1	
dfrA1-like	
dfrA5-14	
dfrA7-17	
dfrA12	
*dfr*S1	
dhfrI	
sul(I)	sulfoxazole (sulfonamide)
sul(II)	
sul(III)	
tap (encoding antibiotic efflux pump)	tetracycline

tetA (encoding Tetracycline Efflux Genes; C: encoding sulfonamide resistance gene)	
tetB (encoding Tetracycline Efflux Genes; C: encoding sulfonamide resistance gene)	
tetB/P (tetracycline resistance gene encoding ribosomal protection proteins)	
tetC (encoding Tetracycline Efflux Genes)	
tetD (Encoding Tetracycline Efflux Genes)	
tetE (Encoding Tetracycline Efflux Genes)	
*tet*Efflux	
tetG (Encoding Tetracycline Efflux Genes)	
tetH (encoding Tetracycline Efflux Genes)	
tetJ (encoding Tetracycline Efflux Genes)	
tetM (tetracycline resistance gene encoding ribosomal protection proteins)	
tetK	
tetL (efflux protein; encoding Tetracycline Efflux Genes)	
tetO (tetracycline resistance gene encoding ribosomal protection proteins)	
tetA(P) (= tetP)	
tetQ (tetracycline resistance gene encoding ribosomal protection proteins)	
tetS (tetracycline resistance gene encoding ribosomal protection proteins)	
tetT (tetracycline resistance gene encoding ribosomal protection proteins)	
tetV (efflux protein)	
tetW (tetracycline resistance gene encoding ribosomal protection proteins)	
tetX	
tetY (encoding Tetracycline Efflux Genes)	
tetZ (encoding Tetracycline Efflux Genes)	
tet30 (encoding Tetracycline Efflux Genes)	
tet32	
tet40	
tet44	
otrA (tetracycline resistance gene encoding ribosomal protection proteins)	

otrB (efflux protein)	
	quinolone
aac(6')-Ib-cr (plasmid-mediated quinolone resistance)	
parC	
qnrA, *qnrB*, *qnrC*, *qnrD* (plasmid-mediated quinolone resistance)	
qnrS (plasmid-mediated quinolone resistance)	
gyrA	
qepA (fluorquinolone efflux pump; plasmid-mediated quinolone resistance)	
oqxA-*oqxB*	
emrA	
emrB	
emrR	
mfd	
cdeA	fluorquinolone
oqxAB (plasmid-encoded antibiotic efflux pump; meaning oqxA and oqxB)	tetracycline, nitrofuran, diaminopyrimidine, fluoroquinolone, glycylcycline
oqxA	
oqxB	
ermA	macrolide, streptogramin, lincosamide
ermB	
ermC	
Inuc	
macB	
mefA (macrolide efflux protein)	macrolide (e.g. erythromycin)
lnuB (*linB)*	lincosamide
lnuC (*linC*)	
msrA	streptogramin, tetracycline, pleuromutilin, macrolide, oxazolidinone, lincosamide, phenicol
vgaA	
vgaA-B	
vgbA	
vgbA-B	
optrA (ABC transporter gene)	
fexA (encoding phenicol exporters)	Antibiotic efflux pump
fexB (encoding phenicol exporters)	

rpoB	rifamycin
vanA	glycopeptide (e.g. vancomycin)
vanB	
vanC	
vanC1	
vanC2	
vanS	
vanX	
vanZ	
fosB	fosfomycin
*fosB*Plasmid	
vatA	streptogramines
vatB	
*vga*AMB	
flo$_{st}$	chloramphenicol, florfenicol
RosB	fosmidomycin
KsgA	kasugamycin
sphAI	other/unknown
dfrA12-aadA2 (gene cassette)	
AAC(6')-Ip	
blaBLEE (aminoglycoside, quinolone)	

Table S9. *Antibiotic resistance genes (ARGs) investigated in included studies and corresponding resistance.*

**Mobile elements**	
**Encoding gene**	**Mobile element type**
intI1	class 1 integrase (→ class 1 integron)
5'CS-3'CS (for detection of class1 integron)	
unknown	
int1 (encoded by genes in integron 1)	aminoglycoside, tetracycline, chloramphenicol, florfenicol, ampicillin and other drugs if resistance gene inserted into integron 1
qacEΔI-sulI (encoded by genes in integron 1)	Quarternary ammonium compounds, sulfonamides

intI2	class 2 integron
mcr-1	Colistin resistance encoding element (mobile resistance gene)
Tn916	Transposable element (transmission of resistance genes; conjugative transposon)
Tn1546	Transposon (Tn3 derivative containing vanA operon)

Table S10. *Mobile genetic elements investigated in included studies.*

Genotypic resistance: Antimicrobial resistance genes represented in included studies (quantitative analysis)

**Antimicrobial resistance genes**		
**Antimicrobial resistance (mechanism) encoded**	**Antimicrobial resistance encoding gene**	**Number of studies (n)**
Beta lactamase/Beta lactams	*amp*C	1
	blaCARB	2
	blaCTX	1
	blaCTX-M	10
	blaCTX-M-1	3
	blaCTX-M-2	2
	blaCTX-M-8	3
	blaCTX-M-9	2
	blaCTX-M-15	1
	blaCMY (plasmid-mediated AmpC (pAmpC))	4
	blaCMY-2 (ceftiofur, ceftriaxone resistance)	5
	blaNDM (carbapenem resistance)	2
	blaNDM-1 (carbapenem resistance)	2
	blaTEM (resistance to ampicillin/amoxicillin)	21
	blaOXA	7
	blaOXA-1	2
	blaOXA-1-like	1
	blaOXA-23	1
	blaOXA-24	1
	blaOXA-58	1
	blaOXY	2
	bla_1 SVH prec	1
	blaPSE(-1) (resistance to ampicillin)	5
	blaSHV	12
	blaSHV-2	1

	blaSHV-5	1
	blaACC (family-specific plasmid-mediated AmpC beta-lactamase gene)	2
	cfxA	1
	CIT (family-specific plasmid-mediated AmpC beta-lactamase gene)	1
	DHA (family-specific plasmid-mediated AmpC beta-lactamase gene)	1
	EBC (family-specific plasmid-mediated AmpC beta-lactamase gene)	1
	FOX (family-specific plasmid-mediated AmpC beta-lactamase gene)	1
	MOX (family-specific plasmid-mediated AmpC beta-lactamase gene)	1
	kpc precursor	1
	smeA	1
	mrdA	1
	blaA	
	blaB	
	*bla*VEB	1
	*bla*GES	2
	*bla*PER	1
	*bla*VIM (carbapenem resistance)	1
	*bla*IMP (carbapenem resistance)	1
	*bla*KPC	1
	many different beta-lactamase genes (e.g. cfxA, blaOXA, blaCTX-M, blaKPC, blaGES (these examples were counted here))	1
	unknown	1
Class A beta-lactamase	*bla*TEM-1	2
beta lactams (PBP-2a)	mecA	2
ampicillin	*pbp5*	1
multidrug resistance	mdfA	1
	mdtA (aminocoumarin resistance)	1
	mdtA-1	1
	mdtB (aminocoumarin resistance)	2
	mdtB-1	1
	mdtB-2	1
	mdtC (aminocoumarin resistance)	2
	mdtC-2	1
	mdtE	1
	mdtF	1
	mdtG	1
	mdtK	1

	mdtM	1
	mdtN	1
	mdtO	1
	mdtP	1
	robA	1
	AcrA	1
	AcrB	1
	AcrE	1
	AcrF	1
	baeR	1
	baeS	1
	Bcr	1
	cpxA	1
	cpxR	1
	CRP	1
	EmrD	1
	evgS	1
	unknown	1
aminocoumarin	alaS	1
	cysB	1
	acrD	1
aminoglycosides	*aac(3)IIa*	2
	aacA-aphD	1
	aacA4 (aminoglycoside acetyltransferase)	1
	aadA1 (=aadA=ant(3'’); ant(3'’)-Ia; streptomycin resistance; gene cassette)	7
	aadA2 (streptomycine resistance; gene cassette)	4
	aadD	1
	aadE	1
	apmA	1
	aphA (aminoglycoside phosphotransferase)	1
	aphA3 (aminoglycoside phosphotransferase)	1
	aph(2”)-Ia-acc(6’)-Ie	1
	aph(2”)-Ib (= aph(2'’)-IIa)	2
	aph(2”)-Ic	1
	aph(2”)-Id	1
	aph(3)IIa	1
	aph(3’)-III	2
	aph(3’)IIIa	4
	aph(3”)-IIIa	1
	aac6	1
	aac(3)-IV	2
	aac(3)-IIa	1
	aac(6’)-Ib (aminoglycoside N-acetyltransferase conferring resistance to tobramycin, kanamycin and amikacin)	5 (of which 1 *“aac(6')-Ib* and its variants (IbL, Ib, others)”)

	AAC(6')-Ie-APH(2'')-Ia (aminoglycoside acetyltransferase encoded by plasmids and transposons)	3
	aac(6')-Ie/aph(2")	1
	ant(2'')-Ia (= aadB)	2
	ant(3")-Ia *(aadA)*	3
	ant(4', 4'')	
	ant(4')-Ia	3
	ant(6)Ia (*aad(6)*)	4
	ANT(6)-Ib	2
	ANT(9)-Ia (aad(9))	1
	neo (aminoglycoside phosphotransferase)	1
	npmA	1
	rmtB (16S rRNA methyltransferase)	1
	strA (streptomycin resistance; = *aph(3'')-Ib*)	4
	strB (streptomycin resistance; = *aph(6)-Id*)	4
	strA-strB (streptomycin resistance)	4
	str (?)	1
	kdpE	1
	tolC	1
	unknown	1
chloramphenicol	cat1 (chloramphenicol acetyltransferase)	2
	cat2 (chloramphenicol acetyltransferase)	1
	*cat(*pC221*)* (A-7) (chloramphenicol acetyltransferase)	1
	*cat(*pC223*)* (A-8) (chloramphenicol acetyltransferase)	1
	*cat(*pC194*)* (A-9) (chloramphenicol acetyltransferase)	1
	cat86 (A-6) (chloramphenicol acetyltransferase)	1
	catS (A-12) (chloramphenicol acetyltransferase)	1
	catDP (A-11) (chloramphenicol acetyltransferase)	1
	catB (A-15) (chloramphenicol acetyltransferase)	1
	catQ (A-16) (chloramphenicol acetyltransferase)	1
	mdtL	1
	flo	1
	floR	3
	cml	1
	cmlA	1
	cmlA1	1
	cmlB	
	catA	1
	catA1	2
	cat3	
phenicol, oxazolidinone, lincosamid, macrolide, streptogramin	*cfr*	2
	cfr1	
	cfr(B)	1

(Cfr rRNA methyltransferase)		
mupirocin	*mupA*	1
	unknown	1
polymyxin	arnA	1
	arnC	1
	arnT_2 (polymyxin B)	1
	arnE (polymyxin B)	1
	mcr-1	5
	PmrB	1
	PmrC	1
	PmrE	1
bacitracin	bacA	2
	bacA_1	1
	uppP	1
streptogramin	*sat* (e.g. sat-4)	1
novobiocin	hslJ	1
streptomycin	*rpsL*	1
sulfonamides	*leuO*	1
	sul1	4
	sul2 (sulfamethoxazole)	5
	sul3	2
	sul(A)	1
	unknown	1
gentamicin (aminoglycoside), kanamycin	*aacC2*	1
	aacC4	1
kanamycin	aphA1	3
	aphA2	1
	Kn	1
trimethoprim	dfrA	1
	dfrA1	2
	dfrA1-like	1
	dfrA5-14	1
	dfrA7-17	1
	dfrA12	2
	*dfr*S1	1

	dhfrI	2
	unknown	1
sulfoxazole (sulfonamide)	*sul(I)*	5
	sul(II)	5
	sul(III)	3
tetracycline	tap (encoding antibiotic efflux pump)	1
	tet (?)	1
	tetA (encoding Tetracycline Efflux Genes; C: encoding sulfonamide resistance gene)	12
	tetB (encoding Tetracycline Efflux Genes; C: encoding sulfonamide resistance gene)	10
	tetB/P (tetracycline resistance gene encoding ribosomal protection proteins)	2
	tetC (encoding Tetracycline Efflux Genes)	9
	tetD (Encoding Tetracycline Efflux Genes)	3
	tetE (Encoding Tetracycline Efflux Genes)	2
	*tet*Efflux	1
	tetG (Encoding Tetracycline Efflux Genes)	7
	tetH (encoding Tetracycline Efflux Genes)	2
	tetJ (encoding Tetracycline Efflux Genes)	2
	tetM (tetracycline resistance gene encoding ribosomal protection proteins)	9
	tetK	6
	tetL (efflux protein; encoding Tetracycline Efflux Genes)	6
	tetO (tetracycline resistance gene encoding ribosomal protection proteins)	7
	tetA(P) (= tetP)	3
	tetQ (tetracycline resistance gene encoding ribosomal protection proteins)	3
	tetS (tetracycline resistance gene encoding ribosomal protection proteins)	4
	tetT (tetracycline resistance gene encoding ribosomal protection proteins)	3
	tetV (efflux protein)	1
	tetW (tetracycline resistance gene encoding ribosomal protection proteins)	3
	tetX	2
	tetY (encoding Tetracycline Efflux Genes)	3
	tetZ (encoding Tetracycline Efflux Genes)	2
	tet30 (encoding Tetracycline Efflux Genes)	2
	tet32	1
	tet40	2
	tet44	1
	otrA (tetracycline resistance gene encoding ribosomal protection proteins)	2
	otrB (efflux protein)	1
	unknown	1

quinolone	*aac(6')-Ib-cr* (plasmid-mediated quinolone resistance)	7
	parC	3
	qnr (?)	1
	qnrA (plasmid-mediated quinolone resistance)	9
	qnrB (plasmid-mediated quinolone resistance)	8
	qnrC (plasmid-mediated quinolone resistance)	4
	qnrD (plasmid-mediated quinolone resistance)	4
	qnrS (plasmid-mediated quinolone resistance)	9
	gyrA	6 (of which 1 determination of mutations in the QRDR of the gyrA gene)
	qepA (fluorquinolone efflux pump; plasmid-mediated quinolone resistance)	7
	oqxA-oqxB (oqxAB)	1
	emrA	1
	emrB	1
	emrR	1
	mfd	1
	unknown	1
fluorquinolone	cdeA	1
tetracycline, nitrofuran, diaminopyrimidine, fluoroquinolone, glycylcycline	oqxAB (plasmid-encoded antibiotic efflux pump; meaning oqxA and oqxB)	1
	oqxA	3
	oqxB	3
macrolide, streptogramin, lincosamide	*ermA*	3
	ermB	5
	ermC	2
	Inuc	1
	macB	1
macrolide (e.g. erythromycin)	*mefA* (macrolide efflux protein)	2
	unknown	1
lincosamide	lnuB (*linB)*	2
	lnuC (*linC*)	1
streptogramin, tetracycline, pleuromutilin, macrolide, oxazolidinone, lincosamide, phenicol	*msrA*	1
	vgaA-B	1
	vgaA	1
	vgbA-B	1
	vgbA	1
	optrA (ABC transporter gene)	1
Antibiotic efflux pump	*fexA* (encoding phenicol exporters)	1

	fexB (encoding phenicol exporters)	1
rifamycin	*rpoB*	1
glycopeptide (e.g. vancomycin)	*vanA*	6
	vanB	4
	vanC	1
	vanC1	1
	vanC2	1
	vanS	1
	vanX	1
	vanZ	1
	unknown	1
fosfomycin	*fosB*	1
	*fosB*Plasmid	1
	unknown	1
streptogramines	*vatA*	1
	vatB	1
	*vga*AMB	1
chloramphenicol, florfenicol	flo_{st}	1
fosmidomycin	RosB	1
kasugamycin	KsgA	1
multiple ARGs (e.g. metagenomic analysis)		6
nitrofurantoin	unknown	1
rifampin	unknown	1
triclosan	unknown	1
streptothiricin	unknown	1
amphenicol	unknown	1
other/unknown	sphAI	1
	dfrA12-aadA2 (gene cassette)	1
	AAC(6')-Ip	1
	blaBLEE (aminoglycoside, quinolone)	1

Table S11. *Antibiotic resistance genes (ARGs) investigated in included studies (n) and corresponding resistance.*

Mobile elements		
**Mobile element type**	**Encoding gene**	**Number of studies (n)**
class 1 integrase (→ class 1 integron)	*intI1*	4
	5'CS-3'CS (for detection of class1 integron)	1
	unknown	6
aminoglycoside, tetracycline, chloramphenicol, florfenicol, ampicillin and other drugs if resistance gene inserted into integron 1	int1 (encoded by genes in integron 1)	1
Quarternary ammonium compounds, sulfonamides	*qacEΔI-sulI* (encoded by genes in integron 1)	1
class 2 integron	*intI2*	3
	unknown	2
Colistin resistance encoding element (mobile resistance gene)	*mcr-1*	5
Transposable element (transmission of resistance genes; conjugative transposon)	*Tn916*	1

Table S12. *Mobile genetic elements investigated in included studies (n).*

Number of studies investigating phenotypic or/and genotypic resistance

Genotypic antibiotic resistance was investigated in a total of 67 out of the 89 studies included in this review. And in 71 phenotypic resistance of the isolates was determined. 49 studies investigated both genetic and phenotypic resistance.
Studies that used isolates that were characterized for phenotypical or genotypical resistance before (not in present study) were not counted here.

5. Methods for antimicrobial susceptibility testing: Phenotypic vs. genotypic resistance, PCR, metagenomic study, whole genome sequencing, MIC, …

Phenotypic resistance

Phenotypic antimicrobial susceptibility testing			
**Method**	**Further differentiation**	**Example/ explanation**	**Number of studies (n) applying this method**
**Culture-based methods**			
Culture (on agar with supplementation of ABC)		Growth on screening plates with colistin	4
Disc diffusion method	Kirby-Bauer = agar diffusion method		44
	modified Kirby-Bauer		1
Dilution methods	Agar dilution method		13
	Broth microdilution method		16
	Automated microdilution system	Automated 96-well based microdilution system Micronaut-S.	1
E-test	= E-strip method = Epsilometer-test		10
**Automated antimicrobial susceptibility testing**			
Vitek®2 system			2
Sensititre			1
MicroScan® system			1
Epsilometer test		E.g. for determination of MIC values for ciprofloxacin	
**Tests for (extended-spectrum) beta-lactamase producers**			
Combined disc diffusion method	For phenotypical confirmation of beta-lactamase producers	Isolates resistant to either ceftazidime or ceftriaxone	1
Double disc synergy test	For phenotypic ESBL production/ expression.<br>= double disc diffusion method		13
Cefinase test	For testing penicillinase production		1
E-test extended spectrum beta lactamase strips	For ESBL production		1

Table S13. *Methods for Phenotypic antimicrobial susceptibility testing*

Genotypic resistance

Genotypic resistance was assessed using PCR-based methods in most cases using specific primers for certain antimicrobial resistance genes (ARGs) (e.g. PCR followed by sequencing). Sometimes – e.g. in metagenomic studies – genome sequences were screened for certain sequences known to be encoding ARGs.
In some cases there were additional tests performed (see appendix III) such as e.g. investigating transferability of antimicrobial resistance genes (e.g. plasmid analysis, filter mating, conjugation assays).

6. Methods for investigation of the direction of transmission/spread: phylogeny, …

The most common method for assessing genetic relatedness and clonality among isolates was pulsed-field gel electrophoresis (PFGE) following digestion with restriction enzymes. Other methods such as multilocus sequence typing (MLST) and whole genome analysis were applied much less.

But only very few studies investigated genetic relations between isolates specifically referring to antimicrobial resistance (e.g. ARGs), and even less the potential spread and direction of spread between the three compartments.

For some methods see appendix III.

Appendix

Appendix I: List of analysis methods for antimicrobial resistance in studies included

Phenotypic resistance

- Culture on agar with ABC (e.g. growth on screening plates with colistin; n=4)

- Disc diffusion (n=39)
- Disc diffusion method (by Kirby-Bauer; n=3)
- Disc diffusion method (by modified Kirby-Bauer; n=1)
- Combined disc diffusion method (e.g. isolates resistant to either ceftazidime or ceftriaxone) for phenotypical confirmation of beta-lactamase producers (only where beta-lactamase production is indicated as possible mechanism explaining observed resistance); n=1)
- Oxoid discs (n=1)
- Broth microdilution method (n=13)
- Automated 96-well based microdilution system Micronaut-S. (n=1)
- Agar dilution method (n=13)
- Agar diffusion methods with antibiotic disks (BD, Sparks, MD; n=1)
- Microbroth method (n=1)
- Microbroth dilution (n=2)
- PCR for detection of resistance genes (n=1)
- Double disc synergy test (ESBL production, n=6)
- Double disc diffusion/ method (ESBL expression; n=7)
- Cefinase test (beta-lactamase/penicillinase production; n=1)

- E-strips method (n=2)
- E-test (n=7)
- E-Test extended spectrum beta lactamase (ESBL) strips (n=1)
- Vitek®2 system for in vitro antibiotic resistance testing (n=2)
- Sensititre automated antimicrobial susceptibility system (phenotypic; MICs) (n=1)
- MicroScan® system (Siemens AG, Munich, Germany) for antimicrobial susceptibility to additional antibiotics and MICs (n=1)

- Epsilometer test (for determination of MIC values for ciprofloxacin; n=1)
- Filter mating, stability of antibiotic resistance (500 bacterial generations) (n=1)
- Filter mating (n=1)

Genotypic resistance

- Whole Genome Sequencing (WGS, e.g. Illumina-HiSeq; n=4)
- Multilocus Sequence Typing (MLST; n=3)
- Southern hybridization (e.g. for presence of AmpC beta-lactamase enzyme encoding gene blaCMY-2; n=1)

- PCR-based replicon typing (e.g. for characterisation of conjugative plasmids or for plasmid analysis (e.g. size, content)) (n=2)
- Multiplex PCR assay (e.g. for *mecA* gene, for detection of genes encoding beta-lactamases) (n=6)

- Modification of 2 multiplex PCR and sequencing (molecular characterization of beta-lactamase genes) (n=1)
- PCR (assays/amplification) for genotypic resistance profiling/ detection of ARGs/plasmids/integrons (n=29)
- PCR-based novel in-house method to screen for presence of *mcr-1*-bearing organisms (n=1)
- PCR (amplification) and sequencing (e.g. for detection and characterization of ABR, for analysis of co-located resistance genes, integrons and *bla*SHV-12 genetic environment, for characterization of class 1 and class 2 integrons) (n=10)
- PCR and sequence analysis (n=1)
- PCR amplification, DNA sequencing and cloning (n=1)
- PCR and conjugation experiments for identification and localisation of resistance genes (n=1)
- PCR-RFLP (n=2)
- PCR-overlapping scheme (Woodford et al.) for determination of backbone structure of Tn1546 (n=1)
- PCR mapping and further sequencing (e.g. of fragments with unusual size) (e.g. for determination of backbone structure of Tn1546 (vanA)) (n=1)
- PFGE for clonality analysis and conjugation assays (n=1)
- Plasmid typing (S1-PFGE/hybridization; n=1)
- Real-time PCR method (e.g. newly established for detection of mcr-1 gene; n=2)
- RFLP analysis (n=3)
- Amplification (e.g. PCR), sequencing and hybridization of gene sequences (ARGs) (n=2)

- Microarray system (check-MDR) (n=2)

- Metagenomic analysis (n=2)
- Using preexisting available whole-genome data (n=1)
- Detection of ARGs through comparison to CARD database with ARIBA software
- BLASTX (e.g. for identification of ARG-like ORFs (automatical sorting into 25 "ARG types" (e.g. tetracycline resistance genes) and 620 "ARG subtypes" (e.g. tetA, tetB, tetC) using a package of customized scripts) or for identification of sequence reads for homology search between metagenomic sequence reads and antibiotic resistance proteins with Antibiotic Resistance Database as reference) (n=2)
- megaBLAST (for comparison of reads from metatranscriptome datasets with genes from resistance determinants database (RED-DB)) (n=1)

- Sequencing (e.g. of amplified integron gene cassettes) (n=2)
- Sequencing of ESBL-genes using Sanger sequencing (n=1)
- DNA sequencing & analysis (n=1)
- Sanger sequencing (n=1)

- Investigation of plasmid acquired genes coding for resistance (n=1)
- S1/I-CeuI hybridization for characterization of plasmids (e.g. size, content) (n=2)
- Method of Barton hybridizing S1 nuclease-digested genomic DNA with a vanA probe following standard procedures (for assessment of plasmid location of vanA genes) (n=1)
- Non-radioactive single-strand conformation polymorphism (SSCP) analysis and DNA sequencing (e.g. for analysis of mutations within quinolone resistance determining region of gyrA gene; n=1)

Appendix II: List of additional analysis methods for antimicrobial resistance in studies included

- Transferability of antimicrobial resistance genes (ARGs) (n=14):
  - In-vitro filter matings/conjugation experiments (n=2)
  - Broth and filter mating assays for assessment of horizontal gene transfer testing for selected vancomycin resistant enterococci with vanA gene (potential inter- and intraspecies horizontal transfer of vancomycin resistance genes) (n=1)
  - Investigation of genetic elements (mobile genetic elements (e.g. aadA5-dfrA17-carrying class 1 integron/ identification of integron-carrying ACCs) and metal resistance genes) (n=1)
  - Investigation of presence and relative abundance of 17 known mobile genetic elements (n=1)
  - Detection of transposable element Tn916 (only phenotypically Tet-resistant isolates; 76 soil and 30 clinical isolates) (n=1)

  Plasmids (n=8)

  - S1-PFGE for plasmid profiles and transferability (n=1)
  - Plasmid transferability (n=1)
  - Plasmid/resistance gene transfer analysis (strain conjugation) (n=1)
  - Search for plasmids following PBRT scheme targeting replicons of the major incompatibility group (Inc) harboring/disseminating ARGs in *Enterobacteriaceae* (n=1)
  - Characterization of plasmids by replicon typing and restriction digestion (n=1)
  - Conjugation experiments and plasmid analysis (transferability of oqxAB genes) (n=1)
  - Study of plasmid-encoded resistance genes (plasmid-mediated quinolone resistance (qnrA, qnrS, aac(6')-Ib-cr, qepA) (n=1)
  - Plasmid characterization by rep-PCR typing (n=1)

- SCCmec typing (n=2):
  - Test for SCCmec typing genes (n=1)
  - Test for SCCmec typing genes using multiplex PCR assay (n=1)

- Xbal-PFGE for investigating genetic diversity (n=1)
- Characterization of *optrA* genetic environment (n=1)
- Investigation of genetic environment of blaCTX-M genes by PCR and sequencing (n=1)
